# Icariin as a potential anticancer agent: a review of its biological effects on various cancers

**DOI:** 10.3389/fphar.2023.1216363

**Published:** 2023-06-30

**Authors:** Fang-Yuan Liu, Dan-Ni Ding, Yun-Rui Wang, Shao-Xuan Liu, Cheng Peng, Fang Shen, Xiao-Ya Zhu, Chan Li, Li-Ping Tang, Feng-Juan Han

**Affiliations:** ^1^ The First Affiliated Hospital of Heilongjiang University of Chinese Medicine, Harbin, China; ^2^ First Clinical Medical College, Heilongjiang University of Chinese Medicine, Harbin, China; ^3^ Harbin Medical University Cancer Hospital, Harbin, China

**Keywords:** icariin, anticancer agent, human cancers, mechanism research, complementary alternative medicine

## Abstract

Numerous chemical compounds used in cancer treatment have been isolated from natural herbs to address the ever-increasing cancer incidence worldwide. Therein is icariin, which has been extensively studied for its therapeutic potential due to its anti-inflammatory, antioxidant, antidepressant, and aphrodisiac properties. However, there is a lack of comprehensive and detailed review of studies on icariin in cancer treatment. Given this, this study reviews and examines the relevant literature on the chemopreventive and therapeutic potentials of icariin in cancer treatment and describes its mechanism of action. The review shows that icariin has the property of inhibiting cancer progression and reversing drug resistance. Therefore, icariin may be a valuable potential agent for the prevention and treatment of various cancers due to its natural origin, safety, and low cost compared to conventional anticancer drugs, while further research on this natural agent is needed.

## 1 Introduction

**FIGURE 1:**
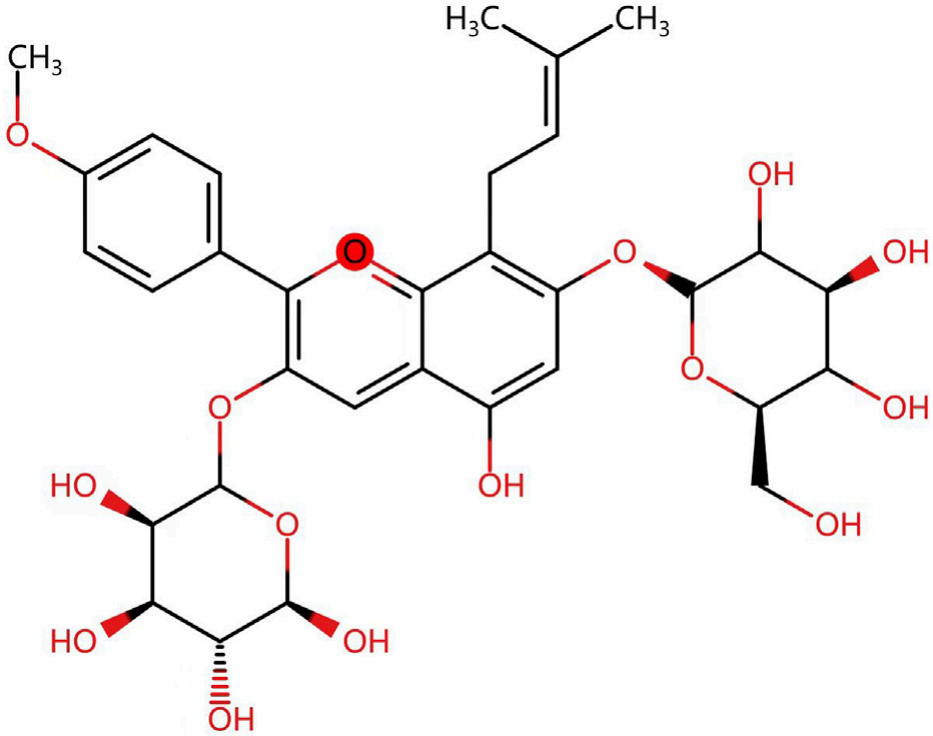
Chemical structure of Icarrin.

*Herba Epimedium*, as one of the representative Chinese medicinal herbs, is described in “Shen Nong’s Herbal Classic” first published in the Han dynasty (202BC-220). Icariin, a class of isoprenoid flavonoids consisting of a glucose group at C-3, a methoxy group at C-4, an isoprenoid group at position C-8, and a rhamnose group at C-7 (Figure 1) (Sun et al., 2019; He et al., 2020), is the primary active constituent of the extract of *Epimedium* (Angeloni et al., 2019). It has been shown to have anti-inflammatory, antioxidant, antidepressant, and aphrodisiac effects (Szabó et al., 2022). In addition, with the advances in modern pharmacological research, the bioactive effects of icariin and its metabolites on cancers (Song et al., 2020), the immune system (Wu et al., 2022), the cardiovascular system (Zeng et al., 2022), the skeletal system (Wang Z. et al., 2018) and other aspects have been gradually discovered.

Cancer remains a global burden despite the technological and pharmaceutical improvements in the last two decades (Sung et al., 2021). Current standard therapies for cancer include surgery, chemotherapy, radiation therapy, targeted therapy, and immunotherapy, which have demonstrated good clinical efficacy but are limited by acquired resistance and severe side effects (Neoptolemos et al., 2018; Nussinov et al., 2021). For many years, complementary and alternative medicine (CAM) has been a valuable therapy for preventing and treating cancers (Xu et al., 2021). Many reports have shown that Chinese medicine offers various preventive and therapeutic options for multiple types of cancers (Wang et al., 2020b; Wang Y. et al., 2020; Zhang et al., 2021; Xu et al., 2022). In recent years, icariin has attracted the attention of the scientific and medical community owing to its anticancer properties, low cost, and few adverse effects. Icariin is generally believed to reduce specific sensitizing and adverse effects when used in combination with other chemotherapeutic agents (Song et al., 2020). In this sense, the impact of icariin on cancer cells has been the subject of research. Tan and Zhang et al. (Tan et al., 2016; Zhang et al., 2020) investigated the mechanisms by which icariin and its derivatives treat various types of cancers, such as induction of apoptosis, regulation of autophagy, and inhibition of angiogenesis. Another work (Liu et al., 2023b) focused on the mechanism of action of icariin in a limited number of cancers. More importantly, however, there needs to be a more thorough and detailed review of all previous studies on icariin in various cancers.

**FIGURE 2:**
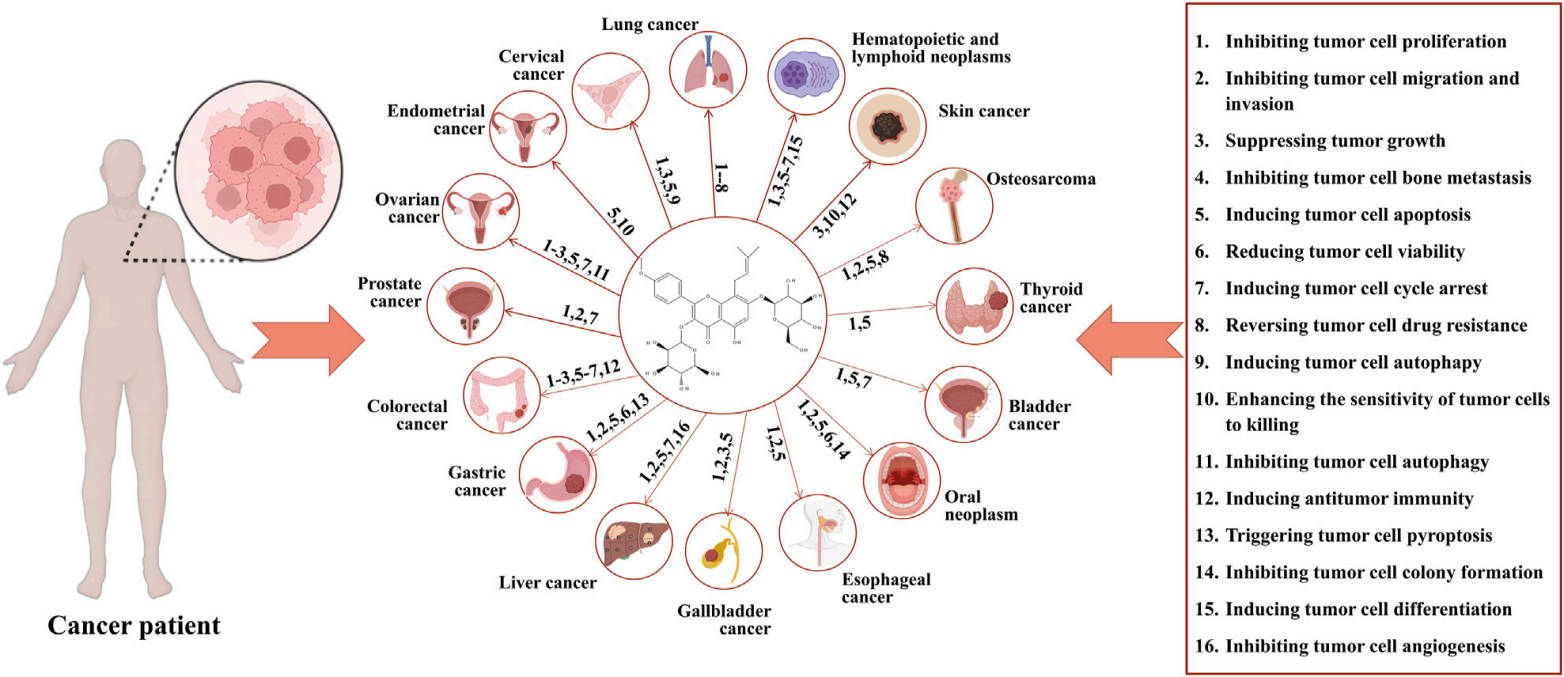
Anticancer mechanisms of icariin. Icariin exerts its anticancer effects via the following actions: 1. inhibition of proliferation of tumor cell; 2. inhibition of migration and invasion of tumor cell; 3. Suppression of tumor growth; 4. inhibition of bone metastasis of tumor cell; 5. induction of apoptosis of tumor cell; 6. reduction of viability of tumor cell; 7. induction of cycle arrest of tumor cell; 8. reverse of drug resistance of tumor cell; 9. induction of autophagy of tumor cell; 10. enhancement of sensitivity of tumor cell to killing; 11. inhibition of autophagy of tumor cell; 12. induction of antitumor immunity; 13. trigger of pyroptosis of tumor cell; 14. inhibition of colony formation of tumor cell; 15. induction of differentiation of tumor cell; 16. inhibition of angiogenesis of tumor cell. The corresponding anticancer actions of icariin in various cancers are as follows: lung cancer: 1–8; cervical cancer: 1.3.5.9; endometrial cancer: 5.10; ovarian cancer: 1.2.3.5.7.11; prostate cancer: 1.2.7; colorectal cancer: 1.2.3.5.6.7.12; gastric cancer: 1.2.5.6.13; liver cancer: 1.2.5.7.16; gallbladder cancer: 1.2.3.5; esophageal cancer: 1.2.5; oral cancer: 1.2.5.6.14; bladder cancer: 1.5.7; thyroid cancer: 1.5; osteosarcoma: 1.2.5.8; skin cancer: 3.10.12; hematopoietic and lymphoid neoplasms: 1.3.5.6.7.15.

This article provides an updated and comprehensive review of the effects of icariin and its mechanisms of action in a broader range of cancers compared to previous reports. We conduct a thorough comparison between icariin and conventional chemotherapeutic drugs, analyzing their mechanisms of action, efficacy, safety, and cost, to gain a deeper understanding of the potential advantages of icariin that may offer in cancer treatment. Our literature review yields insight into future research directions and the significance of icariin in treating cancer. Furthremore, Table 1 presents a summary of clinical studies related to icariin and its metabolite. Supplementary Table S1 summarizes various types of cancers, the mechanisms of action of icariin on these cancers, and a list of relevant references. At the same time, Figure 2 shows the anticancer mechanism of action of icariin.

**TABLE 1:** Tumor related clinical trials with icariin derivatives.

Cancer	Clinical trial	Date	Phase	Intervention/treatment	Study description
Non-small cell lung cancer	—	2017–2018	—	Drug: Icaritin combined with Paclitaxel + Cisplatin or (Nivolumab/Pembrolizumab) + bevacizumab	Actual Enrollment: 33 participants
				Drug: Paclitaxel + Cisplatin or (Nivolumab/Pembrolizumab) + bevacizumab	Intervention Model: Parallel Assignment
					Primary Outcome Measures: median progression-free survival (mPFS)
					Conclusions: The combination of icaritin with standard regimens has shown certain clinical efficacy and good safety tolerability in the treatment of advanced non-small cell lung cancer Zhou et al. (2019)
Advanced hepatocellular carcinoma	NCT02496949	2015	Phase Ib	Drug: Icaritin	Actual Enrollment: 28 participants
					Intervention Model: Single Group Assignment
					Primary Outcome Measures: To assess safety of icaritin in advanced hepatocellular carcinoma patients (Time Frame: 1–2 years)
					Conclusions: Icaritin was generally well-tolerated without dose limited toxicity (DLT) across tested dose levels. Preliminary durable survival benefits observed in patients with advanced HCC patients. Effect correlated with the drug immune-modulation activity Fan et al. (2019)
Advanced hepatocellular carcinoma	NCT01972672	2015	Phase II	Drug: Icaritin	Actual Enrollment: 70 participants
					Intervention Model: Single Group Assignment
					Primary Outcome Measures: time to progress(TTP) (Time Frame: 1–2 years)
					Conclusions: Icaritin has demonstrated satisfactory clinical safety and immunomodulatory clinical efficacy. Improved OS was observed in the subgroups of advanced HCC patients including PD-L1-positive immune cell expression Sun et al. (2018)
Hepatocellular carcinoma (HCC)	NCT03236636	2017–2019	Phase III	Drug: Icaritin	Actual Enrollment: 312 participants
				Drug: Huachansu	Intervention Model: Parallel Assignment
					Primary Outcome Measures: Overall survival (OS) (Time Frame: 2–4 years)
					Condition: Studies that have been completed but not published
HCC	NCT03236649	2017–2022	Phase III	Drug: Icaritin	Actual Enrollment: 89 participants
	Not yet completed			Drug: Sorafenib Tosylate Tablets	Intervention Model: Parallel Assignment
					Primary Outcome Measures: OS (Time Frame: 1–2 years)
Poor Prognosis HCC	NCT05594927	2022–2025	Phase III	Drug: Icaritin	Estimated Enrollment: 261 participants
	Not yet completed			Drug: Huachansu	Intervention Model: Parallel Assignment
					Primary Outcome Measures: OS (Time Frame: From randomization to death from any cause, assessed up to approximately 24 months)

## 2 Search strategy and selection criteria

We searched the PubMed, Web of Science, Embase, Corchrane Library, CNKI, and VIP databases using “Icariin,” “Tumor,” “Cancer,” and related terms as our key words in the search. Relevant literature published between 1990 and 2023 was analyzed, and the reference lists of the identified studies were also searched. A total of 1,243 articles were identified through the search. Articles that may have been influenced by selection bias, detection bias, reporting bias, and other possible sources of biases were excluded. Ultimately, 124 studies were included, of which 6 were relevant to clinical studies (3 of which were published articles, and 3 were still in the clinical trials). Other articles that were included on animals and cell experiments.

## 3 Anticancer mechanisms of icariin

### 3.1 Lung cancer

As the leading cause of cancer-related mortality worldwide, lung cancer is characterized by drug resistance and poor prognosis (Zhu and Ren, 2022). Traditional first-line chemotherapeutic drugs are frequently accompanied by serious side effects when used to treat tumors (Rawal et al., 2023). Therefore, identifying and developing novel effective chemotherapeutic agents with or without reduced side effects will potentially benefit patients with lung cancer. Among the novel agents, icariin has been shown to be a lung-protective source.

Icariin may inhibit the progression of human lung cancer cells by regulating related signaling pathways. Wen et al. treated A549 lung cancer cells with 0–400 μmol/L icariin and tested their survival rate and found that the survival rate of A549 cells treated with icariin (≥100 μmol/L) significantly reduced in a dose-dependent manner (*p* < 0.05). Further mechanistic studies demonstrated that this inhibitory effect of icariin was associated with the suppression of the PI3K/AKT signaling pathway (Wen et al., 2020). Moreover, icariin activated the mitochondrial signaling pathway and induced apoptosis of A540 lung cancer cells by inhibiting the phosphorylation of AKT (Wu et al., 2019). In a similar vein, it has been shown that icariin promotes the inhibition of lung cancer cell proliferation and migration by activating the CaMKII/JNK signaling pathway (Yang et al., 2020) and the endoplasmic reticulum stress signaling (Di et al., 2015; Wen et al., 2020; Rawal et al., 2023). Icariin also acts against bone metastasis in nude mice with lung cancer by inhibiting the receptor activator of nuclear factor kappa-Β-ligand (RANKL) while increasing osteoprotegerin (OPG) expression (Ruilian et al., 2022). Interestingly, there seems to be a discrepancy in Gong’s experiments. The results showed that icariin did not inhibit nicotine-induced proliferation, invasion, and metastasis of lung cancer cells and did not affect downstream apoptotic signaling pathways (Gong, 2013).

Icariin has been reported to regulate the expression of microRNAs. For example, Zhu et al. investigated the effect of icariin as an anticancer agent on A549 and NCI-H1975 lung cancer cells. They found that icariin could suppress lung cancer progression via the miR-205-5p/PTEN and PI3K/Akt signaling pathways *in vivo* and *in vitro* in a time- and dose-dependent manner. More importantly, icariin had hardly any toxic effect on normal cells (Zhu and Ren, 2022). An experiment by Han et al. also demonstrated the inhibitory effect of the miR-370 signaling pathway on lung cancer via downregulating *PIM1*. Moreover, icariin inhibited proliferation and induced apoptosis in A549 and H358 lung cancer cells, which was associated with the activated miR-370 signaling pathway and downregulation of *PIM1* (Han, 2017).

Icariin can affect the cell cycle and drug resistance of lung cancer cells. Zhu et al. found that different concentrations of icariin (20–50 μmol/L) exhibited an effect on A549 lung cancer cells. These dosages of icariin exerted a series of the impact, including (a) causing a cell cycle arrest in S phase, (b) inducing apoptosis, (c) attenuating the transcription of Cyclin A and its dependent kinase CDK2, (d) reregulating the genes (*P53*, *P21*, and *Bax*), and (e) downregulating the gene *Bcl-2* (Zhu, 2013). In terms of chemoresistance, Wu et al. conducted a study to ascertain whether icariin could affect the drug resistance of methotrexate (MTX) -resistant A549 lung cancer cells. They discovered that the half maximal inhibitory concentration (IC_50_) of MTX against A549/MTX-resistant lung cancer cells decreased from 52.17 ± 2.25 μmol/L to 35.50 ± 1.85 μmol/L under the effect of icariin, indicating that icariin can reverse the drug resistance of MTX-resistant A549 lung cancer cells. In another study, icariin was also found to inhibit A549/MTX cell invasion and migration by inhibiting *c-myc* while activating the nm23-h1 mRNA expression (Wu et al., 2009).

Deglycosylation of icariin leads to icaritin, a new active monomer obtained by enzymatic conversion of icariin (Tan et al., 2016). In a clinical trial involving 33 patients with advanced non-small cell lung cancer (Zhou et al., 2019), the median progression-free survival (mPFS) results showed that the median survival was significantly higher in the Icaritin group than that in the conventional treatment group, while the overall response rate (ORR) and disease control rate (DCR) were higher than those in the conventional treatment group but not statistically significant. The two groups was not significantly different in the incidence of adverse reactions. The study suggests that treatment with acalabrutinib in combination with conventional therapies has certain clinical efficacy for advanced non-small cell lung cancer and is safe and well-tolerated (Table 1).

Recently, a novel bionic-targeted icariin nano-preparation has been developed to increase its solubility, utilize its biocompatibility, and improve its capability of tumor penetration, resulting in a better therapeutic effect on lung cancer (Ji et al., 2022).

### 3.2 Cervical carcinoma

Cervical cancer, a malignant tumor that develops in the cervical canal and the vaginal part of the cervix, is one of the most common malignancies in women (Szymonowicz and Chen, 2020). Chemotherapy combined with platinum-based agents and paclitaxel is the dominant treatment for primary cervical cancer and perhaps also the only choice for patients with recurrent cervical cancer. However, the adverse effects of radiotherapy on the bladder, small intestine, and rectum can significantly impact the patients’ quality of life. Moreover, platinum-based (Pt (II)) chemotherapy is inflicted by insufficient non-specific resistance and non-specific dose-dependent toxicity (Jiang et al., 2021). Based on this, the existing literature suggests that icariin can be effective against cervical cancer by regulating the relevant genes and signaling pathways, inhibiting proliferation and promoting apoptosis of TC-1 cells in a time- and concentration-dependent manner (Du et al., 2011). For example, Yu et al. found that the upregulation of chemokine (CXC) receptor 3-B and downregulation of CXC ligand 4 inhibited cell proliferation and differentiation (Yu et al., 2021). During their research, Huang et al. (Huang et al., 2019) discovered that targeting the mTOR/PI3K/AKT signaling pathway can induce apoptosis and autophagy to inhibit the growth of human cervical cancer cells. Additionally, icariin was found to inhibit the growth of human cervical cancer cells by impairing the TLR4/MyD88/NF-κB and Wnt/β-catenin signaling pathways, suppressing inflammation, proliferation, migration, and invasion of CC, and promoting apoptosis *in vivo* and *in vitro* (Li C. et al., 2021).

### 3.3 Endometrial carcinoma

Endometrial carcinoma (EC), or uterine body carcinoma, is a group of malignant epithelial tumors that occur in the endometrium and are among the top three malignant tumors in women (Siegel et al., 2021). Carboplatin plus paclitaxel is the current standard chemotherapy for first-line management of advanced, recurrent and metastatic endometrial carcinoma. Unfortunately, many patients relapse after this treatment. While hormonal therapy is an alternative for hormone-receptor positive tumors, most patients require additional systemic treatment (Vistad and Bjørge, 2023). The present review found that icariin can induce apoptosis and enhance cell killing in EC cells. Zhao et al., for example, exposed B-MD-C1 (ADR^+^/^+^) cells to icariin at various concentrations (12.5/25/50 nmol/L) and found that icariin inhibited the growth of B-MD-C1 (ADR^+^/^+^) cells, which may be related to the fact that the cells were blocked in S phase so that the DNA of B-MD-C1(ADR^+^/^+^) cells could not be adequately replicated in S phase, which eventually lead to inhibition of cell proliferation. Moreover, icariin at lower concentrations cannot inhibit tumor cell growth but can induce the expression of cell surface-associated adhesion molecules and make them more sensitive to cytokine-induced killing by killer cells, which provided an experimental basis for combining icariin with immunotherapy (Zhao et al., 2011). Additionally, icariin-Ⅱ, a metabolite of icariin, has been found to exhibit cytotoxicity in human EC cells. Interestingly, it can also be produced by intestinal fungi (Wang et al., 2022).

### 3.4 Ovarian cancer

Ovarian cancer (OC), a common malignant tumor of the female reproductive system, has an incidence of about 2.4%–5.6% and a mortality rate that ranks first among gynecologic malignancies (Jammal et al., 2017). Most advanced patients may experience recurrence within 3 years after their initial surgical and chemotherapy treatments. Management of recurrance has become increasingly difficult to due to drug resistance, and as a result, the 5-year survival rate has not improved virtually over the past decade. Therefore, there is an urgent need for a treatment that can both consolidate therapy and prolong disease control to improve patient survival rates (Elyashiv et al., 2021). In recent years, much attention has been paid to the role of traditional Chinese herbal medicines in the treatment of OC. Icariin is one of the herbal medicines. Literature of previous studies has shown that icariin can affect the development of OC by regulating relevant signaling pathways, genes, cytokines, and apoptotic proteins. Specifically, icariin can inhibit proliferation, migration, and invasion of OC cells by upregulating microR-519d (Li J. W. et al., 2015), inhibiting Wnt/β-catenin signaling (Chen R. et al., 2019) and NF-κB signaling (Gao et al., 2022), downregulating MMP-2 and MMP-9 (He, 2016; Jiang et al., 2018), and regulating PI3K Akt signaling (Wang et al., 2020a). Upon further investigation, Li et al. (Li J. et al., 2015) found that the molecular mechanisms of such regulation may be related to the targeting of phosphatase and tensin homolog (*PTEN*), *RECK*, and *Bcl-2* genes by microRNA-21.

Meanwhile, icariin can increase the sensitivity of human cell line SKVCR with multidrug-resistant phenotype OC to cisplatin by increasing the proportion of cells in G phase, activating Akt/mTOR signaling pathway, and inhibiting the cell cycle and autophagy (Jiang et al., 2019). In addition, optimized icariin can induce apoptosis and increase cytotoxicity in OC cells by increasing reactive oxygen species (ROS), caspase-3, *p53*, and TNF-α (Alhakamy et al., 2020). Interestingly, another study (Wang et al., 2019) found that icariin did not affect apoptosis and autophagy in SKOV3 cells but only inhibited cell cycle transition and expression of fuse binding protein 1 (FBP1) and β-catenin to suppress viability, colony, formation, and migration of OC cells. Experiments in a xenograft mouse model by Fu et al. (Fu et al., 2022) demonstrated that icariin significantly attenuated ovarian tumor growth for the first time. They also revealed that icariin attenuated tumor progression by suppressing TNKS2/Wnt/β-catenin signaling by upregulating the level of *miR-1-3p* in OC with transcriptome analysis.

### 3.5 Prostate cancer

Prostate cancer (PC), the second most common cancer in men with the fifth highest cancer mortality rate (Bray et al., 2018), is a highly androgen-dependent disease (Desai et al., 2021). Androgen-deprivation therapy (ADT) is commonly used as the first-line therapy for the treatment of advanced prostate cancer. However, this therapy is associated with numerous adverse reactions that cannot to be overlooked (Lafontaine and Kokorovic, 2022). Once the disease progresses to castration-resistant prostate cancer (CRPC), docetaxel is the main remaining drug that has been proven to provide a survival benefit in metastatic CRPC. Unfortunately, docetaxel has some cytotoxic effects such as neutropenia, anemia, and thrombocytopenia that should not be disregarded (Buttigliero et al., 2018). Icariin has been found to be able to affect the progression of PC (Gu et al., 2020). The mechanisms by which icariin interferes with the progression of PC include (a) inhibition of androgen receptor activity, which binds androgens and induces activation of target genes such as prostate-specific antigen (PSA) to induce PC (Zhang, 2013; Zhang et al., 2017; Chen D. S. et al., 2018); (b) inhibition of expression of key markers of PC, PSA, and inhibition of tumor growth (Zhang, 2013; Chen S. X. et al., 2018); (c) increase of E-cadherin, decrease of calcitonin, and increase of adhesion between tumor cells and stroma and inhibition of PI3K/AKT signaling to suppress migration and invasion of PC cells (Chen S. X. et al., 2018); (d) arrest of cell cycle (Chen D. S. et al., 2018; He et al., 2019); and (e) inhibition of PC tumor vascular remodeling and migration by inhibiting MMPs and the Notch-1 pathway (Zhang et al., 2017).

Rao et al. found that icariin can inhibit the growth of foregut adenocarcinomas *in vivo*, possibly by inhibiting fatty acid synthase (FAS) from reducing the synthesis of endogenous fatty acids needed for tumor cell growth and by slowing down the uncontrolled proliferation of malignant tumors in S phase, thus inhibiting their growth, migration, and invasion (Rao et al., 2018). Furthermore, most studies have shown that icariin can arrest the cell cycle by blocking cells in S phase (Chen S. X. et al., 2018; He et al., 2019). Another study found that icariin could not induce apoptosis by blocking the cycle in G0/G1 phase (Zhang, 2013). Thus, further studies have yet to be conducted to cope with this discrepancy.

### 3.6 Colorectal cancer

Colorectal cancer (CRC) is a common malignant tumor of the digestive system with high morbidity and mortality. Surgical treatment remains the most effective treatment for CRC, while preoperative adjuvant chemoradiotherapy is also widely employed (Zhang et al., 2014). Due to the increasing resistance to conventional medicine, new therapies should be urgently explored (Tian et al., 2018). Since it can effectively inhibit the migration and viability of CRC cells and promote apoptosis (Tian et al., 2018; Zhang et al., 2019), icariin has been shown to have an antitumor effect on CRC in a *p53*-dependent manner (Tian et al., 2018). Furthermore, it can enhance the antiproliferative effect of radiation on cancer cells by inhibiting the expression of NF-κB, which arrests the cell cycle in G2/M phase (Zhang et al., 2014). For example, Kim et al. showed that icariin (10 μM) could make CRC cells sensitive to *TRAIL*-induced apoptosis by downregulating cell survival proteins while upregulating cell apoptosis protein expression, and inducing the expression of DR4 and DR5 by activating the ROS-ERK-CHOP pathway (Kim et al., 2020). As it can also act as a chemotherapeutic sensitizer for colon cancer cells, icariin (20 µM) can enhance the antitumor activity of 5-FU in colon carcinomas by inhibiting NF-κB activity (Shi et al., 2014). The combination of icariin (100 nM) with cisplatin (4 µM) showed more effective antitumor activity than treatment with either icariin or cisplatin alone (Tian et al., 2018). In addition, icariin can induce antitumor immunity in a CD8 T-cell-dependent way (Hao et al., 2019).

### 3.7 Gastric cancer

Gastric cancer (GC) is the fifth most common cancer and the third most common cause of cancer death worldwide (Smyth et al., 2020). Due to the poor prognosis for patients with advanced GC, there has been an urgent need to develop new strategies to improve survival in this disease (Zhao et al., 2019). As a messenger of cellular delivery, Ca^2+^ plays an important role in cell proliferation, differentiation, metastasis, and invasion (Tiffner and Derler, 2020). Icariin can inhibit proliferation and induce apoptosis of GC SGC-7901 cells by upregulating the expression of calcium-sensing receptor (CaSR) and runt-related transcription factor 3 (*RUNX3*) and inhibiting survival (Li, 2016). Similarly, icariin can also inhibit GC cell progression via multiple mechanisms, such as (a) regulating the hsa_circ_0003159/eIF4A3/bcl-2 axis to promote GC cell apoptosis (Yin et al., 2022); (b) regulating the hsa_circ_0003159/miR-223-3p/NLRP3 signaling axis to inhibit GC cell viability and trigger cell pyroptosis (Zhang F. et al., 2022); (c) blocking the cell cycle in G0/G1 phase (Duan et al., 2009); and (d) downregulating metastasis-related proteins (Chen S. H. et al., 2019). In addition, icariin can inhibit tumor cell invasion and migration via the Rac 1-dependent VASP pathway (Wang et al., 2010).

### 3.8 Liver cancer

Primary liver cancer is the fifth most common cancer worldwide and the third leading cause of death. Hepatocellular carcinoma (HCC) is the most common form of primary liver cancer. Nearly 600,000 people die from this disease worldwide every year (Sung et al., 2021). Although for a small number of early-stage patients, surgical resection, liver transplantation or transarterial chemoembolization are feasible options, most patients with primary liver cancer are in the advanced stage and are not suitable for surgical treatment (Rizvi et al., 2021). Currently, sorafenib, lenvatinib and donafenib are among the first-line medication for systemic treatment of these patients but their efficacy is far from desirable (Mokdad et al., 2016; Li et al., 2023a). With this in mind, a number of studies have been conducted in search of such therapies. Icariin is one of the subjects of such studies. Studies have shown that icariin exerts an anti-tumor effect on liver cancer cells in a concentration-dependent manner. Its mechanisms of action can be summarized as follows: (a) inhibition of proliferation of tumor cell (Bi et al., 2022; Xu and You, 2023), (b) induction of apoptosis of tumor cell (Zhu et al., 2012; Li, 2014; Bi et al., 2022; Xu and You, 2023), (c) induction of arrest of tumor cell cycle in G1 to S phase (Bi et al., 2022) or G0 to G1 phase (Zhu et al., 2012; Xu and You, 2023), (d) suppression of adhesion and migration of tumor cell (Wang and Peng, 2011), (e) inhibition of angiogenesis of tumor cell (Yang et al., 2009).

From the perspective of combination therapy, Li (2014) study suggests that icariin can enhance the anti-tumor activity of arsenic trioxide, which may be associated with the generation of intracellular ROS and the inhibition of NF-κB activity. It has been found that the combination of icariin with docetaxel can lower the expression levels of April and VEGF in HepG2 cells, thereby inhibiting the growth of endothelial cells (ECV304) (Tang et al., 2009). Additionally, icariin can inhibit the apoptosis of HepG2-induced T lymphocytes, enhance the cytotoxic sensitivity of CD3AK cells, and reverse the immune escape of tumor cell (Tang et al., 2007; Wang et al., 2007). Ding et al. (2023) used water-soluble trace polymers to synthesize with HP-γ-cyclodextrin to increase the water solubility of icariin by 654-fold. They showed that when the icariin complex is used, fewer drugs are need for the same therapeutic effect, which can be used potentially as a potent hepatoprotective agent or anti-liver cancer treatment drug.

Given that icaritin is an important derivative of icariin, Li et al. conducted a synthesis and structure-activity analysis of icaritin and validated its potential as a tumor growth inhibitor for liver cancer cells (Li et al., 2023b). They also found that the tumor suppressing factor HBP1 can inhibit the migration and invasion of liver cancer cells by suppressing the AFP promoter, and HBP1’s expression is associated with the malignancy of liver cancer (Cao et al., 2021). Studies have shown that icaritin can enhance the inhibitory effect of HBP1 by strengthening its binding to the AFP promoter (Cao et al., 2021; Li C. et al., 2021), lowersing MMP9 levels (Cao et al., 2021), and increasing p53 protein expression (Li H. et al., 2021), thereby reducing the viability and migration of HepG2 cells and inducing apoptosis as well. In addition, Xue et al. (2023) demonstrated in *in vitro* and *in vivo* experiments that treatment with icaritin significantly reduced tumor size and had a potential anti-proliferative effect on HCC cells. They further analyzed and speculated that tyrosine-protein kinase Fyn may could possibly be used as a key target.

In recent years, Shenogen Pharma (Beijing, China) has developed icaritin either as a monotherapeutic drug or a combo-drug combined with an oncolytic virus, primarily for the treatment of liver cancer (Bailly, 2020). Their icaritin-derived drugs have undergone several clinical trials. The results of Phase I trials (NCT01278810) showed that icaritin has good safety and tolerability. The bioavailability of icaritin is high after meals, and its half-life is relatively short. Phase Ib clinical trials (NCT02496949) (Fan et al., 2019) showed that icaritin has good safety and preliminary long-term survival benefits for patients with advanced HCC. These results suggest that icaritin has the potential to serve as a new oral immunotherapy for advanced HCC, in addition to the existing antibody-based PD-1/PD-L1 blockade therapy. Additionally, phase II clinical trial with icaritin (NCT01972672) (Sun et al., 2018) in advanced HCC has been completed. The ORR in this trial was comparable to that of the existing clinical trial data for current treatments. The median overall survival (OS) of the study population was 179 days, which was slightly longer than the median OS of Sorafenib Tosylate Tablets and FOLFOX regimens in Chinese population trials (175 days and 171 days, respectively). A phase III clinical trial is currently underway comparing icaritin with Sorafenib Tosylate Tablets and Huachansu for the treatment of liver cancer (NCT03236636, NCT03236649, NCT05594927).

### 3.9 Gallbladder cancer

Gallbladder cancer (GBC) is a rare disease associated with gallstones and chronic gallbladder inflammation. It is insidious because it is usually not diagnosed until at an advanced stage when the tumor is large enough to cause obstruction and invade adjacent structures (Baiu and Visser, 2018). Surgical resection is still the only treatment with curative intent for GBC, but very few cases are suitable for such resection. Treatment options for patients with inoperable GBC include radiotherapy and systemic chemotherapy (Roa et al., 2022). Clinical trials have demonstrated both the efficacy and safety of gemcitabine in treating patients with GBC. However, since the response rate to gemcitabine is not satisfactory, new treatment strategies are needed to improve response rates and prolong survival (Zhang et al., 2013).

It has been reported that icariin can induce apoptosis of GBC cells and arrest the cell cycle. For example, icariin (40–160 μg/mL) has been shown to suppress cell proliferation and induce apoptosis in both GBC-SD and SGC-996 cells in a dose-dependent manner. For another example, icariin (40 μg/mL) with gemcitabine has been demonstrated to enhance caspase-3 activity and suppress the expression of Bcl-2, Bcl-xL, and surviving proteins, thus inhibiting NF-κB activity *in vivo* and *in vitro*. Icariin can also enhance the cytotoxicity of gemcitabine in GBC-SD cells, while icariin (40 μg/mL) in combination with gemcitabine (0.5 μmol/L) can induce arrest of G0-G1 phase, thereby inhibiting cell proliferation (Zhang et al., 2013). For example, Zhang et al. found that icariin significantly increased homotypic adhesion in GBC-SD cells, inhibited heterogeneous adhesion between GBC-SD cells and the matrix, and suppressed invasion of GBC-SD cells. More importantly, icariin has little effect on the viability of normal cells and therefore has no systemic toxicity (Zhang et al., 2010).

### 3.10 Esophageal cancer

Esophageal cancer is the seventh most common malignant tumor worldwide and has the sixth highest cancer-related mortality rate (Bray et al., 2018). At present, the prognosis of advanced esophageal cancer (EC) is far from desirable, and few effective emedies are available. For EC patients in advanced stage, a combination of platinum and fluoropyrimidine is recognized as the standard first-line therapy (Ikeda et al., 2022). The aim of chemotherapy is to alleviate symptoms and improve survival. Although some symptomatic improvement can be achieved in patients with the use of standard first-line chemotherapy regimens, response rates are usually low and short lasting (Jin et al., 2009). Literature from previous studies shows that icariin can induce apoptosis and inhibit the proliferation, migration, and invasion of esophageal cancer cells. Icariin has been evidenced to slow down the growth rate of esophageal cancer cells *in vivo*. *In vitro*, icariin can inhibit the proliferation of TE-13 and A109 esophageal cancer cells, the mechanism of which may be related to the upregulation of Fasl, Fas, and interferon-γ expression (Ji et al., 2016). The inhibitory effect of icariin is possibly associated with the induction of apoptosis by ROS- mediated downregulation of mitochondrial membrane potential, P-AKT, P-STAT3, P-85, and KI67 expression, as well as cell cycle arrest in G2/M phase. Icariin can also inhibit cell migration and invasion by upregulating the expression of epithelial markers and downregulating the mesenchymal marker expression (Gu et al., 2017). For example, Fan et al. found that icariin exerts an anticancer effect by activating the pro-apoptotic pathway mediated by endoplasmic reticulum stress (ERS). Icariin can activate ROS by increasing nicotinamide adenine dinucleotide phosphate (NADPH) oxidase activity, increasing the expression of the pro-apoptotic protein (PUMA) and the levels of ERS-related molecules (GRP78, ATF4, CHOP, p-PERK, and p-eIF2α), and decreasing the expression of intracellular glutathione (GSH), Bcl-2 and caspase-9 *in vitro* and *in vivo* (Fan et al., 2016). In addition, since icariin, one of the active ingredients of *Epimedium*, can increase the expression of Hedgehog, Smo and Gli, and glycogen synthase kinase 3β (GSK3β) as well as decrease Wnt and β-catenin levels, it can induce apoptosis and inhibit the proliferation of CD133 + cells (Han, 2019).

### 3.11 Oral neoplasm

As the 6th most common cancer worldwide, oral neoplasms constitute a significant health problem, and their prevalence is on the rise (Masthan et al., 2012). First-line therapies for oral cancer typically involve surgery, radiation therapy, and in certain cases, chemotherapy. Radiation and chemotherapy agents, such as cisplatin and 5-fluorouracil (5-FU), primarily exert their cytotoxic effects on cancer cells by inducing DNA damage. However, despite the initial response to treatment, tumor recurrence and acquired resistance remain as significant clinical challenges, with 5-year survival rates being only 10%–20% for stage III and IV patients. Therefore, it is urgent to develop novel therapeutic approaches and combinations to overcome drug resistance in oral (Wang et al., 2021). Icariin has anticancer effects on oral neoplasms by inhibiting NF-κB and PI3K/AKT signaling pathways, thereby inhibiting cell proliferation and migration and inducing apoptosis (Lei et al., 2020; Sun and Zhang, 2021). Icariin has been shown to inhibit NF-κB and PI3K/AKT signaling pathways by inhibiting the expression of anti-apoptotic protein Bcl-2, upregulating the expression of Caspas3 protein, and downregulating the expression of p-p65 and p-AKT, thereby inhibiting the proliferation of oral squamous cell carcinoma (OSCC) cells and inducing apoptosis (Sun and Zhang, 2021). It can also block NF-κB signaling by inhibiting Toll-like receptor 4 (*TLR4*). The latter is a member of TLRs, which are mediators indispensable to innate immunity and can recognize a considerable number of inflammatory triggers. Once activated, TLRs can recruit MyD88 (an adaptor molecule) to activate the NF-kB signaling pathway. In addition, studies have revealed that icariin can suppress OSCC cell viability, colony formation, and invasion, which may be related to the inhibition of the TLR4/NF-κB pathway (Lei et al., 2020).

### 3.12 Bladder cancer

Bladder cancer (BC) is one of the most common urologic malignancies, and its incidence rate is increasing worldwide (Lenis et al., 2020). Currently, there is no oral medication available that can effectively reduce recurrence and progression in patients with BC (Seok et al., 2023). *Bacillus* Calmette-Guerin immunotherapy has been traditionally used to decrease the rate of recurrence of with Non-muscle-invasive BC patients (Brausi et al., 2014). However, this therapy may have some potential side effects, such as high fever, bladder discomfort, and urinary tract infections. Moreover, for patients who do not respond to immunotherapy, therapeutic options are limited, and the prognosis for their recurrence remains unfavorable (Brausi et al., 2014). Icariin is reported to exert a significant adverse effect on the progression and invasion of BC by inhibiting the proliferation of BC cells and increasing apoptosis. Specifically, icariin can induce apoptosis in T24 cells, which may be related to the downregulation of Bcl-2, upregulation of Bax, and arrest of the cell cycle in G0/G1 phase (Gao, 2010). For example, Kang et al. demonstrated that icariin could inhibit proliferation and induce apoptosis of BIU87 cells by regulating the *GRP78* gene at the gene level (Kang et al., 2013).

### 3.13 Thyroid cancer

Thyroid cancer (TC), a malignant tumor of the endocrine system, accounts for about 1% of all tumors worldwide, while an alarming increase in its incidence has been observed in recent years. Differentiated thyroid cancer, as the most common histological subtype, can typically be cured through surgery and radioactive iodine (RAI) therapy (Roof and Geiger, 2023). RAI is the first-line therapy for patients with metastatic papillary thyroid carcinoma. Unfortunately, 60% of patients become resistant to RAI therapy and develop radioactive-iodine refractory disease, eventually leading to an overall poor prognosis (Liu et al., 2023a). Studies in recent years have shown that icariin plays a unique role in the treatment of thyroid cancer. Bisphenol A (BPA), an endocrine-disruptive chemical, may be positively associated with TC (Alsen et al., 2021). However, although BPA can promote the proliferation of human TC B-CPAP cells and reduce apoptosis, the process is susceptible to icariin and thus can be reversed by the latter. The mechanism underlying this may be associated with the induction of oxidative damage and activation of apoptosis by increasing the expression of intracellular ROS and inhibiting the expression of antioxidant enzyme systems (Zheng et al., 2017).

### 3.14 Osteosarcoma

Osteosarcoma is the most common primary malignancy of the bone and has a high propensity for local invasion and metastasis. The primary treatment modalities for it are surgery and radiotherapy (Chen et al., 2021). However, as azithromycin is one of the most commonly used chemotherapeutic agents for osteosarcoma, tumor cell resistance after chemotherapy has become a significant clinical problem. In such a context, icariin was found to be able to suppress the growth ability of MG-63 and MG-63/DOX cells and increase their sensitivity to azithromycin, while upregulating the expression of cleaved caspase-9, cleaved caspase-3, and cleaved PARP, enhancing azithromycin-induced apoptosis, downregulating the expression of the transporter protein MDR1 expression, and reversing the multidrug resistance of azithromycin (Wang et al., 2015a). In a recent study, Wang et al. (Wang Z. D. et al., 2018) found that icariin could serve as a potential agent to reverse MDR by inhibiting the expression of MDR1 and MRP1 and suggested that its mechanism to regulate multidrug resistance may be related to blocked STAT3 phosphorylation.

Icariin also plays a vital role in promoting apoptosis and inhibiting proliferation and invasion of human osteosarcoma cells (Lu, 2012; Ren et al., 2018). Detection of related gene expression by semi-quantitative polymerase chain reaction revealed that icariin treatment could significantly decrease the expression levels of β-catenin, c-Myc, cell cycle protein D1, MMP-9, and vascular endothelial growth factor (VEGF) while increasing the expression of caspase-3 in osteosarcoma cells 143B. The results suggest that icariin suppresses the expression of the Wnt/β-catenin signaling pathway and target proteins related to it via inhibiting the phosphorylation of p-GSK3β, thereby regulating the proliferation and invasion of osteosarcoma cells (Ren et al., 2018). Similar results were provided by the experiments of Tan (Tan and Mai, 2019) et al.

### 3.15 Skin cancer

Skin cancer, by far the most common type of cancer, includes melanoma, basal and squamous cell carcinoma, Merkel cell carcinoma, and lymphoma of the skin (Rauf et al., 2018). Melanoma, the most severe skin cancer, is often considered one of the most aggressive human cancers. Moreover, its high invasiveness and rapid and expansive transmission make surgery an unlikely option (Wu et al., 2015). Although chemotherapy has achieved some effects, it has some limitations and high toxicity. Therefore, it has become urgent to discover novel and less toxic potential candidates for treating melanoma (Wang et al., 2017).

Against this backdrop, icariin has come to the medical community’s attention because of its ability to inhibit the progression of skin cancer by affecting the immune system’s microenvironment and causing cell cycle arrest. For example, Dong et al. found that treatment with icariin and CpG inhibited tumor growth, reversed tumor immunosuppressive microenvironment, and improved the efficacy of immune checkpoint inhibitor therapy in mice with melanoma (Dongye et al., 2022). Furthermore, icariin was found to elicit CD8 T-cell-dependent antitumor immunity. In addition, a treatment combining anti-PD-1/CTLA-4 with icariin significantly improved the antitumor capability and efficacy of either treatment alone (Hao et al., 2019). Furthermore, icariin inhibited the proliferation of melanoma B16 cells and reduced colony formation in a concentration- and time-dependent manner. In addition, icariin can induce the differentiation and cell cycle arrest of melanoma B16 cells at G0/G1 phase by inhibiting the Erk1/2-p38-JNK-dependent pathway (Wang et al., 2017). In an investigation by Li and coworkers, treatment with icariin was shown to inhibit the proliferation of B16 tumor cells in a dose-dependent manner, with an IC_50_ value of 84.3 μg/mL at 72 h (Li X. et al., 2014). Icariside II (IS), a metabolite of icariin, is derived from *Epimedium* (Wang M. et al., 2020). IS has also been found to reduce cell viability and proliferation, increase cell death, and arrest the cell cycle, which is probably associated with the activation of the ROS-p38-p53 signaling pathway (Wu et al., 2015).

Non-melanoma skin cancer (NMSC), consisting predominantly of basal cell carcinoma and squamous cell carcinoma (also called epidermoid carcinoma), is one of the most common malignancies in the United States, with more than two million new cases annually (Rogers et al., 2010; Aggarwal et al., 2021). It was shown that IS could inhibit the cell viability of the A431 cells by regulating apoptosis in a dose-dependent manner. Furthermore, treatment with 50 µm of IS could increase apoptosis of A431 cells by increasing cleaved caspase-9 and cleaved PARP. In addition, treatment with 50 µm of IS can significantly inhibit activation of the Janus kinase (JAK)-STAT3 and mitogen-activated protein kinase (MAPK)-ERK pathways but promote activation of the PI3K-AKT pathway (Wu et al., 2013).

### 3.16 Hematopoietic and lymphoid neoplasms

According to WHO, hematopoietic and lymphoid neoplasms include chronic myeloid leukemia, chronic neutrophilic leukemia, polycythemia vera, primary myelofibrosis, essential thrombocythemia, and chronic eosinophilic leukemia—not otherwise specified as well as myeloproliferative neoplasms (MPN), unclassifiable (MPN-U), *etc.* (Barbui et al., 2018). The results of previous studies suggest that icariin may inhibit tumor growth *in vivo* and *in vitro*, possibly via several mechanisms, which include (a) induction of leukemia cell differentiation (Ge et al., 2001; Li, 2009), (b) apoptosis and arrest of the cell cycle in the G0/G1-phase (Ge et al., 2001; Song, 2012; Song et al., 2015), and (c) synergy with targeted drugs (As2O3 (Wang et al., 2015b) and ATRA (Ge et al., 2001)) and the mechanisms could act via oxidative stress and PI3K/AKT pathway (Pan and Yuan, 2016; Wang and Ke, 2018). In myelodysplastic syndromes (MDS), icariin can increase peripheral hemogram, improve hematopoiesis of morbid bone marrow, and decrease the apoptosis of bone marrow cells in MDS model rats (Feng et al., 2007). Li and colleagues found that icariin inhibited the proliferation of Raji cells by arresting the cell cycle in S phase, inducing the apoptosis of cells associated with activation of caspase-8 and caspase-9 and cleavage of PARP, and decreasing Bcl-2 level, thereby altering the Bcl-2/Bax ratio, and reducing c-Myc (Li Z. J. et al., 2014). However, Lin and colleagues found that icariin failed to inhibit Raji cell growth at any concentrations tested (Lin et al., 2004). Therefore, further research on the impact of icariin on MDS is still needed.

Multiple myeloma (MM), the second most common blood cancer after leukemia, has seen a steady rise in incidence worldwide for the past 30 years (Owens, 2020). Currently, chemotherapy is the primary treatment for MM, with bortezomib/melphalan/prednisone as the first-line medicine (Moreau et al., 2017). However, some MM patients still resist therapy and relapse or are refractory. Therefore, looking for novel and more effective therapeutic agents for MM on a phytochemical basis is urgent. Luckily, icariin has been evidenced to be capable of inhibiting the proliferation of MM cells, inducing apoptosis, and arresting the cell cycle in S phase. Icariin was found to inhibit the proliferation of U266 cells and primary MM cells by inducing S-phase blockade (Zhu et al., 2015). Icariin can also significantly upregulate the expression of Bak and Bax and inhibit the expression of Bcl-xL, phosphoSTAT3 (*p-STAT3*), and phosphor-JAK2 (*p-JAK2*) in a dose-dependent manner. An increased concentration of icariin can cleave and activate caspase 3 and caspase 9. These results suggest that icariin can induce apoptosis of MM cells, and the action is involved in the caspases signaling pathway and the IL-6/JAK2/STAT3 signaling pathway (Zhu et al., 2015; Jung et al., 2018). Icariin was also found to inhibit tumor growth and lower the serum levels of IL-6 and IgE in an MM xenograft mouse model without adverse effects such as weight loss (Zhu et al., 2015). In addition, Li found that icariin induced apoptosis by activating the Erk/JNK pathway (LI, 2011).

## 4 Discussion

As mentioned above, icariin has showing promising anti-tumor activity in various types of cancer. To gain a more in-depth understanding of its potential advantages in cancer treatment, this discussion will examine its mechanisms of action, compare it with conventional chemotherapeutic drugs, and explore its future prospects.

### 4.1 Mechanisms of anti-tumor activity

Based on our review of the mechanisms of actions of icariin and its derivatives against various types of cancers, we make a summary in the following aspects:

(i) Anti-tumor activity: Icariin inhibits the proliferation, invasion, angiogenesis, bone metastasis, autophagy, and colony formation of tumor cells. Additionally, it can suppress tumor cell viability and growth by inducing apoptosis, differentiation, pyroptosis, autophagy, and cell cycle arrest.
(ii) Modulation of the immune system: Icariin and its derivatives may enhance the body’s immune response to cancer by modulating the function of the immune system (Sun et al., 2018; Fan et al., 2019). This response can enhance immune cell activity, promote immune cell proliferation and function, and regulate inflammation (Hao et al., 2019; Lei et al., 2020). They can also modulate immune cells and their secretion of cytokines through multiple pathways, thereby regulating the balance of the immune system and acting directly on immune surveillance, homeostasis, and defense against tumors (Zhao et al., 2011; Hao et al., 2019; Dongye et al., 2022). As a natural immune modulator, icariin has certain advantages compared to synthetic drugs in safety and tolerability. However, given the current limited clinical research on icariin, further evaluation of its efficacy and safety in cancer treatment is badly needed.
(iii) Reversal of drug resistance: Icariin may reverse tumor cell resistance by modulating the expression of drug resistance-related proteins and signaling pathways, accumulating more chemotherapeutic drugs within tumor cells, and enhancing cytotoxicity and sensitivity to cell killing.

### 4.2 Comparison with conventional chemotherapeutic drugs

Icariin has shown promising advantages over conventional chemotherapeutic drugs in cancer treatment. However, these advantages require further research and clinical validation as they not exempt from limitations of their own. Therefore, we conducted a comparative analysis of ICA and conventional chemotherapeutic drugs in terms of their mechanisms of action, efficacy, safety, and cost.

#### 4.2.1 Mechanisms of action

Conventional chemotherapeutic drugs inhibit primarily the growth and division of tumor cells and induce cell death through mechanisms such as anti-metabolism, interaction with DNA, and disruption of microtubule dynamics (Nussbaumer et al., 2011; Guichard et al., 2017; Bukowski et al., 2020). In comparison, ICA may exert its anti-tumor effects through multiple pathways, including inhibiting tumor cell proliferation and migration, inducing tumor cell differentiation and apoptosis, cell cycle arrest, autophagy regulation, inhibition of tumor angiogenesis, and modulation of immune function. These mechanisms and pathways may provide new insights for cancer treatment.

#### 4.2.2 Efficacy

Chemotherapeutic drugs are widely used in clinical treatment and can exert anti-tumor effects on various types of cancer (Devita and Chu, 2008; Ghosh, 2019; Knezevic and Clarke, 2020). Given this, however, currently, there are relatively few researches on the clinical use of icariin in cancer treatment. As of now, only a total of 6 relevant studies have been identified in our literature search (See Table 1 for details). These studies suggest that icariin and its derivatives demonstrated good safety, tolerance, and preliminary durable survival benefits in lung cancer and advanced hepatocellular carcinoma patients. Although the therapeutic efficacy of icariin has not been fully confirmed, some cellular and animal experimental studies have demonstrated that icariin may inhibit the growth, invasion, drug resistance of tumor cells, and induce the apoptosis of tumor cells through multiple pathways. Importantly, icarinn has no toxic side effects on normal cells (Zhu and REN, 2022; Liu et al., 2023b). Additionally, icariin can synergistically enhance the therapeutic effects of conventional chemotherapeutic drugs and increase the sensitivity of cancer cells to chemotherapeutic drugs (Zhai et al., 2021). Therefore, we speculate that icariin may serve as a complementary or adjunctive treatment to traditional chemotherapeutic drugs.

#### 4.2.3 Safety and adverse events

Current clinical studies indicate that ICA has a relatively high safety profile, although it may cause minor adverse reactions such as rash and diarrhea (Fan et al., 2019; Teo et al., 2019; Yuan et al., 2022), which are far-outweighed by its benefits. Furthermore, icariin has the potential to improve immune suppression in cancer patients and provide better therapeutic tolerability (Fan et al., 2019), although further clinical trials are warranted to validate these effects. In comparison, traditional chemotherapeutic drugs often come with more side effects. For example, non-specific chemotherapeutic drugs like cisplatin not only kill tumor cells but also cause systemic toxicity, including nephrotoxicity, neurotoxicity, ototoxicity, and myelosuppression (Zhang C. et al., 2022). Prolonged use of ICA can result in severe damage to normal tissues.

#### 4.2.4 Cost

The cost of icariin typically varies depending on factors such as brand, formulation, and market supply. However, it is generally considered to be relatively affordable, which can potentially alleviate some of the financial burden on patients. On the other hand, chemotherapeutic drugs are frequently linked with higher costs, especially novel targeted therapies and immunotherapies (Pfister, 2013).

### 4.3 Future prospects

**FIGURE 3:**
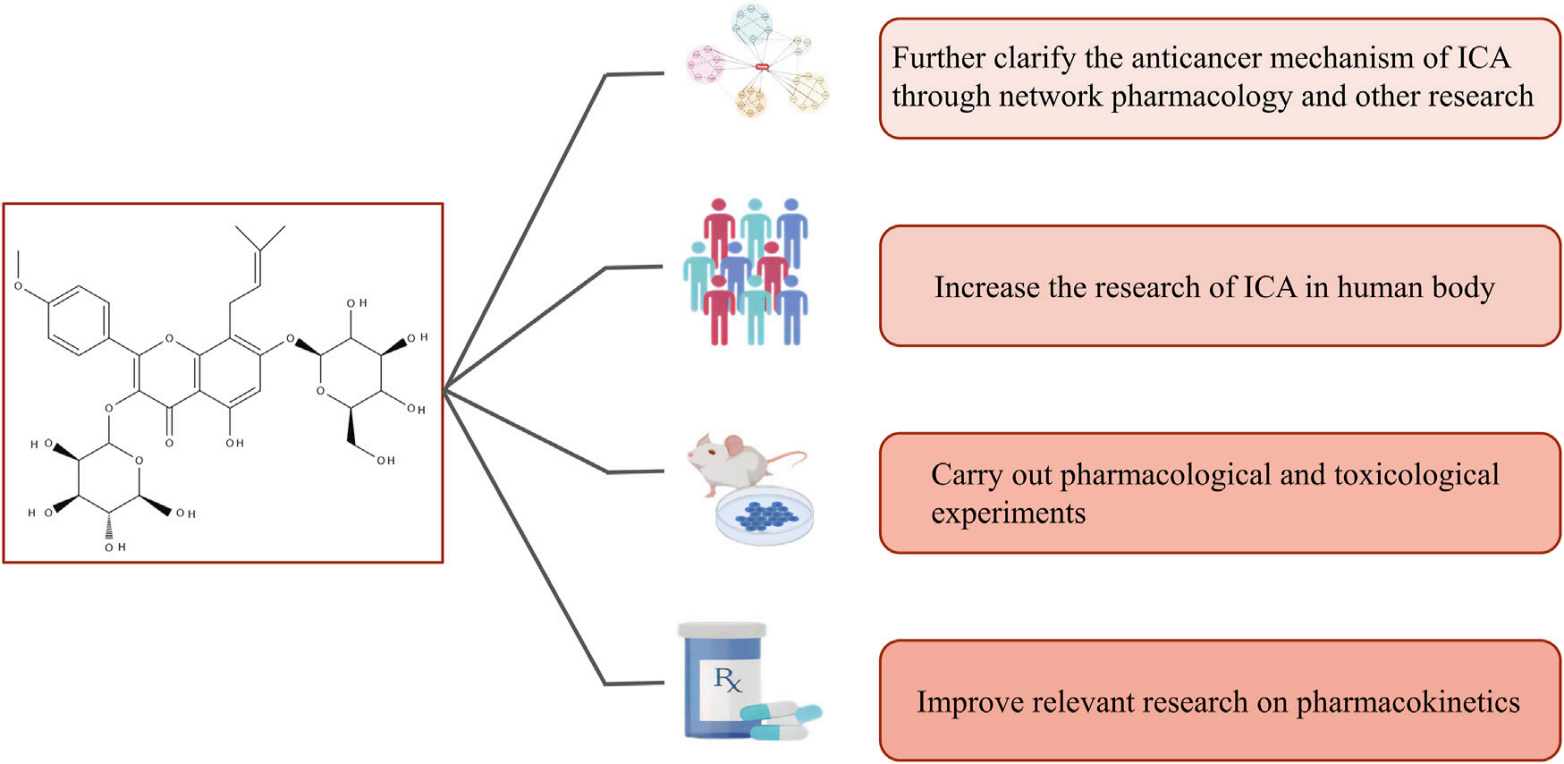
Future in-depth research on the anticancer mechanism of icariin.

As most findings on icariin in the treatment of cancer cited in this review are based on *in vitro* and *in vivo* studies, they are not necessarily representative enough of the effects of icariin on humans alone or in combination with other drugs. Thus, further studies involving various pharmacokinetic parameters are required before the compound is recognized and marketed as a potential prescription anticancer drug. In addition, standardized extracts or dosages should be researched and developed for clinical trials. Therefore, we look forward to future studies on icariin under the following aspects: (a) further clarification of the anticancer mechanism of icariin through network pharmacology and other research, (b) increased research on icariin in humans, (c) conducting further pharmacological and toxicological experiments, and (d) improving relevant research on pharmacokinetics (Figure 3).

In-depth future research on icariin holds significant importance in the following ways. Firstly, it may lead to the discovery of new therapeutic targets. Further research can provide insights into the interactions between icariin and cancer cells of different types. This in turn can help unravel the molecular targets of icariin in cancer and promote the development of novel anticancer drugs. Secondly, it can enhance therapeutic efficacy and improve patients’ quality of life. Future studies can optimize the drug properties, dosage, and administration protocols of icariin to improve its stability and efficacy within the body while minimizing toxic side effects. This in turn can help mitigate or circumvent and ameliorate the adverse reactions associated with conventional chemotherapeutic drugs. Thirdly, it can optimize combination therapy. Future research in this vein can explore the synergistic effects of icariin when it is combined with other anticancer treatment modalities such as chemotherapy, radiation therapy, and immunotherapy. This may facilitate the identification of optimal treatment combinations to enhance therapeutic outcomes and reduce reliance on a single treatment modality. Finally, it can promote personalized approaches to treatment. In-depth research can help determine the efficacy variations of icariin among different patients, cancer subtypes, and molecular subgroups. It can also aid in predicting and monitoring the therapeutic efficacy and adverse reactions of icariin. This can contribute to personalized treatments by identifying the ideal patient population that can optimally benefit from icariin therapy.

## 5 Conclusion

Recent years has witnessed increasing amounts of studies and discussion of the traditional Chinese medicine in medical research for the treatment of tumors. Our review demonstrates that icariin may be a valuable complementary medicine for the prevention and treatment of various cancers due to its natural origin, anti-tumor activity, safety, and low cost compared to conventional cancer drugs. However, there is a lack of sufficient clinical research supporting its exact role and efficacy in the human body. As such, further laboratory research and clinical trials are still needed to validate its safety and effectiveness. We believe that with further in-depth research, icariin has the potential to become a candidate drug for future generations of cancer treatment.

## Figures and Tables

**Table:** 

**5-FU**	5-fluorouracil
**ADT**	Androgen-deprivation therapy
**BC**	Bladder cancer
**BPA**	Bisphenol A
**CAM**	Complementary and Alternative Medicine
**CRC**	Colorectal cancer
**CRPC**	Castration-resistant prostate cancer
**CXC**	Chemokine
**DCR**	Disease control rate
**EC**	Endometrial cancer
**ERS**	Endoplasmic reticulum stress
**FAS**	Fatty acid synthase
**FBP1**	Fuse binding protein 1
**GBC**	Gallbladder cancer
**GC**	Gastric cancer
**GSH**	Glutathione
**GSK3β**	Glycogen synthase kinase 3β
**HCC**	Hepatocellular carcinoma
**IC**_**50**_	Half maximal inhibitory concentration
**IS**	Icariside II
**MDS**	Myelodysplastic syndromes
**MM**	Multiple myeloma
**MPFS**	Median progression-free survival
**MPN**	Myeloproliferative neoplasms
**MTX**	Methotrexate
**NADPH**	Nicotinamide adenine dinucleotide phosphate
**NMSC**	Non-melanoma skin cancer
**OC**	Ovarian cancer
**OPG**	Osteoprotegerin
**ORR**	Overall response rate
**OS**	Overall survival
**OSCC**	Oral squamous cell carcinoma
**PC**	Prostate cancer
**PSA**	Prostate specific antigen
**PTEN**	Phosphatase and tensin homolog
**PUMA**	Pro-apoptotic protein
**RAI**	Radioactive iodine
**ROS**	Reactive oxygen species
**RANKL**	Receptor activator of nuclear factor kappa-Β ligand
**TC**	Thyroid cancer
**TLR4**	Toll-like receptor 4
**VEGF**	Vascular endothelial growth factor
**WHO**	The World Health Organization
