# Supplementary material for: Icariin as a potential anticancer agent: a review of its biological effects on various cancers
Source: Front Pharmacol. 2023 Jun 30;14:1216363. doi: 10.3389/fphar.2023.1216363 (PMC10347417; doi:10.3389/fphar.2023.1216363)
Supplement: Supplementary file 1 [file Table1.docx]

**Supplementary Table S1. Overview of the biological effects of icariin on various types of cancer**

|  | | Agents | Research type | Cell lines / Animal | Targets | Mechanisms | Refs. |
| --- | --- | --- | --- | --- | --- | --- | --- |
| **Lung Cancer** | Icariin | *In vitro* | A549, NCI-H1975 cells | miR-205-5p↓, PTEN↑. | Inhibiting proliferation, migration, and invasion. | ^[20]^ |  |
|  | Icariin | *In vivo* | BALB/C nude mice |  | Suppressing the tumor growth. |  |  |
|  | Icariin | *In vivo* | Nude mice | RANKL↓, OPG↑. | Inhibiting lung cancer bone metastasis, and alleviating osteolytic bone destruction. | ^[24]^ |  |
|  | Icariin | *In vitro* | A549 cells | N-cadherin↓, E-cadherin↑, vimentin↓, PI3K↓, AKT↓, | Inhibiting cell viability and migration. | ^[22]^ |  |
|  | Icariin | *In vitro* | A549 cells | VEGF↓,N-cadherin↓, vimentin↓,  E-cadherin↑, p-CaMKII/CaMKII↑, p-JNK/JNK↑. | Inhibiting proliferation, invasion, and migration. | ^[164]^ |  |
|  | Icariin | *In vitro* | A549, H1299, and H1975 cells | Caspase-3↑, Caspase-9↑, Akt↓, Bax↓, Bad↓, cytochrome C↓, mitochondrial membrane potential↓. | Inhibiting proliferation and inducing apoptosis. | ^[23]^ |  |
|  | Icariin | *In vivo* | Nude mice |  | Inhibiting proliferation. |  |  |
|  | Icariin | *In vitro* | A549 cells | GSH↓, p-PERK↑, ATF6↑, GRP78↑, p-eIF2a↑, CHOP↑, PUMA↑, Bcl2↓. | Inhibiting cell viability and inducing apoptosis. | ^[165]^ |  |
|  | Icariin | *In vivo* | Nude mice | GRP78↑, CHOP↑, PUMA↑, Bcl2↓. | Inhibiting tumor growth. |  |  |
|  | Icariin | *In vitro* | A549,H358 cells | Caspase-3↑,  miR-370↑,  PIM1↓ | Inhibiting proliferation, migration, and invasion, and inducing apoptosis. | ^[26]^ |  |
|  | Icariin | *In vivo* | BALB/C nude mice |  | Inhibiting tumor growth. |  |  |
|  | Icariin | *In vitro* | A549 cells | Cyclin A↓, CDK2↓, P53↑, P21↑, Bcl-2↓, Bax↑ | Inducing cell cycle-arrest in S phase and apoptosis. | ^[27]^ |  |
|  | Icariin | *In vitro* | A549/MTX cells | nm23-h1↑, c-myc↓ | Reversing the metastatic phenotype of A549/MTX resistant cells. | ^[28]^ |  |
| **Cervical Cancer** | Icariin | *In vitro* | Hela cells | CXCR3-B↑, CXCL4↓ | Inhibiting proliferation and differentiation. | ^[34]^ |  |
|  | Icariin | *In vitro* | TC-1 cells |  | Inhibiting proliferation and inducing apoptosis. | ^[33]^ |  |
|  | Icariin | *In vitro* | Hela cells | cleaved caspase-3↑, cleaved caspase-9↑, Bax↑, Bcl-2↓, LC3 II↑, ROS↑, MMP↓, p-PI3K↓, p-Akt↓, mTOR↓ | Inhibiting growth and inducing apoptosis, and autophapy. | ^[35]^ |  |
|  | Icariin | *In vitro and in vivo* | SiHa and U14 cells; Kunming mice | Ki67↓, sunivin↓, Bcl-2↓, c-Myc↓, p16↑, p53↑, Bax↑, serum hemolysin↑, Leukocytes↓ | Inhibiting growth and inducing apoptosis. | ^[36]^ |  |
| **Endometrial Cancer** | Icariin | *In vitro* | B--C1 (ADR + / +) cells | G0/G1↓, S↑,CD54 ↑，CD18↑ | Inhibiting apoptosis and enhancing sensitivity of cell killing. | ^[39]^ |  |
| **Ovarian Cancer** | Icariin | *In vitro* | CAOV3cells | β-catenin↑, c-Myc↓, cyclin D1↓ | Inhibiting proliferation. | ^[44]^ |  |
|  | Icariin | *In vitro* | SKOV3cells | miR-519α↑ | Inhibiting proliferation, migration and invasion. | ^[43]^ |  |
|  | Icariin | *In vitro* | SKOV3 and SKVCR cells | caspase-3↑ | Inhibiting proliferation, migration and invasion, and inducing apoptosis. | ^[47]^ |  |
|  | Icariin | *In vitro* | SKOV3 cells | MMP-2↓, MMP-9↓ | Inhibiting migration and invasion. | ^[46]^ |  |
|  | Optimized Icariin | *In vitro* | SKOV3 cells | ROS↑, p53↑, caspase-3↑, G2/M↑, TNF-α↑ | Inducing apoptosis and enhancing cytotoxicity. | ^[51]^ |  |
|  | Icariin | *In vitro* | SKVCR cells | G0/G1↑, Bax↑, caspase-3↑, LC3B↓, Beclin-1↓, ATG5↓, p62↓ | Inducing apoptosis, blocking cell cycle, inhibiting autophagy, and increasing sensitivity to cisplatin. | ^[47]^ |  |
|  | Icariin | *In vitro* | A2780 cells | caspase-3↑, miR-21↓, PTEN↑, RECK↑, Bcl-2↓ | Inhibiting proliferation and inducing apoptosis. | ^[49]^ |  |
|  | Icariin | *In vitro* | SKOV3 cells | G1↑, cyclinD1↓, cyclinE↓, FBP1↓, c-Myc↓, β-catenin↓ | Suppressing cell cycle transition and cell migration. | ^[52]^ |  |
|  | Icariin | *In vitro and in vivo* | SKOV-3 and HEK293T cells, BALB/C nude mice | TNKS2/Wnt/β-catenin signaling↓, miR-1-3p↑ | Inhibiting proliferation and suppressing tumor growth. | ^[53]^ |  |
|  | Icariin | *In vitro* | SKOV3 cells | Bax↑, Bcl-xl↑, Caspase-3↑, IκBα↓, NF-κBp65↓, p-NF-κBp65↓, p-IκBα↓ | Promoting apoptosis. | ^[45]^ |  |
| **Prostate Cancer** | Icariin | *In vivo* | BALB/c-nu rats | p-AKT↓, p-AK↓, Calctionin ↓, E-cadherin ↑, AR-VT ↓ | Inhibiting cell proliferation and invasion. | ^[61]^ |  |
|  | Icariin | *In vivo* | SCID rats | AR mRNA ↓, p-AR ↓, PSA ↓, PTEN mRNA ↓, G0/G1 ↓, S ↑ | Inhibiting cell proliferation and blocking cell cycle. | ^[60]^ |  |
|  | Icariin | *In vitro* | LNCaP cells | G0/G1 ↑, S ↓, AR ↓, PSA ↓, p-AKT/AKT ↓ | Inhibiting cell proliferation and blocking cell cycle. | ^[58]^ |  |
|  | Icariin | *In vitro and in vivo* | SCID rats and LNCaP cells | G0/G1 ↓, S ↑, FAS ↓, Fas mRNA ↓ | Inhibiting cell proliferation and blocking cell cycle. | ^[63]^ |  |
|  | Icariin | *In vitro* | Du145 and PC3 cells | Notch-1 ↓, MMP-2 ↓, MMP-9 ↓, Hes-1 ↓ | Inhibiting cell proliferation,migration and invasion. | ^[59]^ |  |
|  | Icariin | *In vitro* | PC3 cells |  | Inhibiting cell proliferation. | ^[166]^ |  |
|  | Icariin | *In vivo* | SCID rats | G0/G1 ↓, S ↑, p-AR ↓, AR-mRNA ↓, PTEN mRNA ↑ | Inhibiting cell proliferation and blocking cell cycle. | ^[62]^ |  |
| **Colorectal Cancer** | Icariin / Icariin with Radiation | *In vitro and in vivo* | HCT116 and HT29 cells; Nude mice | NF-κB↓,Bcl-2↓, Bcl-XL↓, VEGF↓, cyclin D1↓, | Inhibiting proliferation，inducing apoptosis, and reducing Tumor volume. | ^[64]^ |  |
|  | Icariin | *In vitro* | HCT116 cells | p-p53↑, p21↑, Bax↑, γ-H2AX↑, Caspase-3↑, Caspase-9↑, Bcl-2 ↓, | Inhibiting migration and viability, and inducing apoptosis. | ^[65]^ |  |
|  | Icariin | *In vitro and in vivo* | HCT116 and HT29 cells; Nude mice | Caspase-3↑, Caspase-9↑, DR4↑, DR5↑, CHOP↑, BAX↑, ROS↑, MAPK signaling↑, ERK↑, Bcl-2↓, Bcl-xL↓, c-IAP-1↓, survivin↓,XIAP↓ | Inducing apoptosis, and Inhibiting tumor growth. | ^[67]^ |  |
|  | Icariin With 5-FU | *In vitro and in vivo* | HT29 and HCT116 cells; Nude mice | NF-κB↓, caspase-8↑, caspase-9↑, caspase-3↑, Bax↑,PARP↑,Bcl-xL↓, cyclin D1↓, | Inhibiting tumor growth and inducing apoptosis. | ^[68]^ |  |
|  | Icariin | *In vitro* | HT29 cells | G0/G1 ↑, G2/M ↑, ALDH2↑, CPNE1 ↑ | Inhibiting cell proliferation, adhesion, and invasion, inducing apoptosis, and blocking cell cycle. | ^[66]^ |  |
|  | Icariin | *In vivo* | CT26 mice | p53 ↑, Bax ↑, Bcl-2 ↓ | Inhibiting tumor growth and inducing apoptosis. | ^[66]^ |  |
|  | Icariin | *In vivo* | C57BL/6 mice | CD8 T-cell↑, | Inducing antitumor immunity. | ^[69]^ |  |
| **Gastric Cancer** | Icariin | *In vitro* | SGC-7901 cells | CasR↑, Runx3↑, Survivin↓ | Inhibiting proliferation and inducing apoptosis. | ^[73]^ |  |
|  | Icariin | *In vitro* | SGC-7901 cells | G0/G1↑, S↓, G2/M↓ | Inhibiting proliferation. | ^[76]^ |  |
|  | Icariin | *In vitro* | MGC803 cells | MMP-2↓, MMP-9↓, MTA2↓ | Inhibiting proliferation, migration and invasion. | ^[77]^ |  |
|  | Icariin | *In vitro* | BGC-823 cells | Rac1↑，VASP↑ | Inhibiting migration and invasion | ^[78]^ |  |
|  | Icariin | *In vitro* | HGC-27 and BGC-803 cells | Bcl-2↓, circ_0003159↑ | Reducing activity and inducing apoptosis. | ^[74]^ |  |
|  | Icariin | *In vitro* | BGC-823 and HGC-27 cells | hsa_circ_0003159↑，miR-223-3p↓, NLRP3↑,caspase-1↑, IL-1β↑, | Inhibiting cell viability and trigger cell pyroptosis. | ^[75]^ |  |
| **Liver**  **Cancer** | Icariin | *In vitro and in vivo* | HepG2 cells | CD31↓, Ki67↓, CD4+↑, CD8+↑, CD19+↑, CD4/CD8↑, | Anti-angiogenesis effects of tumors; anti-proliferating effects on the tumor cells. | ^[87]^ |  |
|  | Icaritin | *In vitro* | HepG2 and SMMC-7721 cells. | G0/G1 phase in the cell cycle↑ | Inducing cell apoptosis | ^[92]^ |  |
|  | Icaritin | *In vitro* | HepG2 cells | AFP↓, AFP mRNA↓, HBP1↑, PTEN↑, caspase3↑, MMP9↓, p-AKT↓, AKT↑ | Inhibiting cell viability and cell migration. | ^[93]^ |  |
|  | Icaritin | *In vitro and in vivo* | HepG2 cells, SMMC7721 cells | p53↑, AFP mRNA↓, AFP↓ | Inhibiting HCC cellular proliferation; inducing HCC cellular apoptosis. | ^[94]^ |  |
|  | Icaritin | *In vitro and in vivo* | HepG2 cells | FYN↑, PIK3R1 ↑ | Anti-proliferative effect. | ^[95]^ |  |
|  | Icariin | *In vitro* | HepG2 cells | CDK4↓, cyclinD1↓, AHR↑, Bax ↑, Bcl-2 ↓ | Inhibiting cell proliferation; inducing apoptosis; blocking cell cycle in G0-G1 phase. | ^[82]^ |  |
|  | Icariin | *In vitro* | CLC5 cells | CDK4↓, cyclinD1↓, p-AKT↓, p-GSK3β ↓ | Inhibiting cell proliferation; blocking cell cycle in G1/S phase. | ^[83]^ |  |
|  | Icariin with arsenic trioxide | *In vitro* | SMMC-7721 and HepG2 cells | ROS↑, NFκB↓, Bcl-2↓, BxL-xL↓, c-myc↓, cyclinD1↓, survivin↓, VEGF↓, | Inhibiting cell proliferation; inducing apoptosis; enhancing the antitumor activity of arsenic trioxide. | ^[85]^ |  |
|  | Icariin | *In vitro* | SMMC-7721 cells | PCNA ↓, Bcl-2 ↓, Bax ↑ | Inhibiting cell proliferation; inducing apoptosis; blocking cell cycle in G0-G1 phase. | ^[84]^ |  |
|  | Icariin | *In vitro* | HepG2 cells |  | Inhibiting HepG2 cell adhesion and migration. | ^[86]^ |  |
|  | Icariin with doxorubicin | *In vitro* | HepG2 cells | APRIL mRNA ↓, VEGF ↓, Bcl-2 ↓, ECV304 cells ↓ | Inhibiting cell proliferation; inhibiting vascular endothelial cell growth. | ^[88, 90]^ |  |
|  | Icariin | *In vitro* | HepG2.2.15 cells | FasL↓, Fas↑ | Inhibiting cell proliferation; reversing immune escape of tumor cells. | ^[89]^ |  |
|  |  |  |  |  |  |  |  |
| **Gallbladder Cancer** | Icariin with gemcitabine | *In vitro* | GBC-SD cells | Caspase-3↑ , Bcl-2 ↓, Bcl-xL ↓, surviving proteins ↓, NF-κB pathway↓ | Inhibiting cell proliferation and inducing apoptosis. | ^[101]^ |  |
|  | Icariin with gemcitabine | *In vivo* | Female BALB/c mice |  | Reducing tumor volume. | ^[101]^ |  |
|  | Icariin | *In vitro* | GBC-SD cells |  | Increasing the homogeneous adhesion, and inhibiting the heterogeneous adhesion, and invasion. | ^[102]^ |  |
| **Esophageal Cancer** | Icariin | *In vitro* and *vivo* | TE-13 and Eca-109 cells | FAS↑, Fasl↑, IFN－γ↑ | Inhibiting proliferation and inducing apoptosis. | ^[105]^ |  |
|  | Icariin | *In vitro* and *vivo* | Kyse70 cells | P-85↓, P-STAT3↓, p-AKT↓, MMP↓, N-cadherin↓, ZEB1↓, Slug↓, ZO-1↑, Ki67↓ | Inhibiting proliferation, migration and invasion, and inducing apoptosis. | ^[106]^ |  |
|  | Icariin | *In vitro* and *vivo* | EC109, TE1, and HET-1A Cells | Caspase-9↑, NADPH↑, GRP78↑, ATF4↑, CHOP↑, p-PERK↑, p-eIF2α↑, PUMA↑, GSH↓, Bcl-2↓ | Inhibiting proliferation, adhesion and migration, and inducing apoptosis. | ^[107]^ |  |
|  | Icariin | *In vitro* | CD133^+^cells | Hedgehog↑, Smo↑, GSK3β↑, Gli↑, Wnt↓, β-catenin↓ | Inhibiting proliferation, migration and invasion, and inducing apoptosis. | ^[108]^ |  |
| **Oral cancer** | Icariin | *In vitro* | Cal 27  and SCC9 cells | cleaved‑caspase‑3↑, pro‑caspase‑3↓, Bax mRNA↑,Bcl-2 mRNA↓, p-p65↓, p-AKT↓, NF‑κB and PI3K/AKT signaling pathways↓ | Inhibiting cell proliferation and inducing apoptosis. | ^[111]^ |  |
|  | Icariin | *In vitro and in vivo* | SCC-9 and SCC-15 cells | MMP-9↓, vimentin↓, p-p65↓, p65↓, ki67↓, PCNA↓, TLR4↓, TLR4/NF-κB signaling pathway↓ | Suppressing the viability, colony formation, and invasion. | ^[112]^ |  |
| **Bladder Cancer** | Icariin | *In vitro* | BIU87 cells | GRP78mRNA↓ | Inhibiting proliferation and inducing apoptosis. | ^[117]^ |  |
|  | Icariin | *in vitro* | T24 cells | Bcl-2↓, Bax↑ | Inhibiting proliferation, and inducing apoptosis and blocking cell cycle. | ^[116]^ |  |
| **Thyroid Cancer** | Icariin with BPA | *In vitro* | B-CPAP cells | ROS↑, MDA↑, SOD↑, γ-H2AX↓, Bcl-2↓ | Inhibiting cell proliferation and inducing apoptosis. | ^[121]^ |  |
| **Osteosarcoma** | Icariin | *In vitro* | U2OS cells | MMP-9↓, cleaved caspase-3↑, Ki67↓ | Inhibiting proliferation and migration, and inducing apoptosis. | ^[127]^ |  |
|  | Icariin | *In vitro* | MG-63 and MG-63/DOX cells | MDR1↓, MRP1↓, STAT3↓, p-STAT3↓ | Reversaling of multidrug resistance. | ^[124]^ |  |
|  | Icariin | *In vitro* | 143B cells | p-GSK3β↓, β-catenin↓, c-Myc↓, cyclin D1↓, caspase-3↓, VEGF↓, MMP-9↓ | Inhibiting proliferation and inducing apoptosis. | ^[126]^ |  |
|  | Icariin | *In vitro* | MG-63 and MG-63/DOX cells | MDR1↓, cleaved caspase-9↓, cleaved caspase-3↓, cleaved PARP↓, p-AKT↓, p-PI3K-p85α↓ | Reversaling of multidrug resistance. | ^[123]^ |  |
|  | Icariin | *In vitro* | MG-63 cells | caspase-3↓, caspase-8↓, caspase-9↓ | Inhibiting proliferation and inducing apoptosis. | ^[125]^ |  |
| **Skin Cancer** | Icariin with CpG | *In vivo and in vitro* | C57BL/6 mice (B16F10 melanoma model), B16F10 cells, | CD8+ T cells↑, CD95↑, CD80↑, MHC-I↑, PD-L1↓, CD8/CD4↑, Th1 cells↑, CTLs↑, CTLs/Tregs↑, DCs↑, TNF-α↑, TAMs↑, | Inhibiting tumor growth, reverting the immunosuppressive microenvironment, prolonging the survival time of melanoma bearing mice, and potentiating the effect of antiPD-1 plus antiCTLA-4 blockade. | ^[131]^ |  |
|  | Icariin | *In vivo* | C57BL/6 mice (B16F10 melanoma model) | CD8 T cells↑,  MDSCs↓, PD-L1↓, | Inhibiting tumor growth and inducing antitumor immunity. | ^[69]^ |  |
|  | Icariin | *In vitro* | B16 cells | MITF↑, Tyr↑, Trp1↑, Trp2↑, CDK2↓, Cyclin A↓, p21↓, Erk1/2-p38-JNK signaling↓, | Inhibiting the proliferation, and arresting cell cycle at G0/G1phase. | ^[130]^ |  |
|  | Icariside II | *In vitro* | A375 human melanoma cells | Cyclin E↓, CDK2↓, cyclin B1↓, P-CDK1↓, ROS↑, p38↑, p53↑, p21↑. | Inhibiting cell viability and proliferation, and inducing cell cycle arrest in G0/G1 phase. | ^[129]^ |  |
|  | Icariin | *In vitro and in vivo* | B16 cells | procaspase-9↓, caspase-9↑, | Inducing B16 melanoma tumor cells apoptosis *in vitro* and inhibiting tumor growth and metastasis *in vivo*. | ^[132]^ |  |
|  | Icariside II | *In vitro* | A431 cells | Cleaved Caspase-9, Cleaved PARP↑, p-AKT↑, p-EGFR↓, p-STAT3↓, p-ERK↓ | Inhibiting cell viability and apoptosis. | ^[136]^ |  |
| **Hematopoietic and Lymphoid Neoplasms** | Icariin | *In vitro* | K562 cells | CD71 ↑, CD235a ↑, P-P38↑, JNK↑/↓ | Inducing cell differentiation. | ^[138]^ |  |
|  | Icariin / Icariin +ATRA | *In vitro* and *vivo* | HL-60/primary APLcells and BALB/C rats | G0/G1-phase ↑, S-phase ↓ | Inducing cell differentiation and inducing apoptosis and blocking cell cycle. | ^[139]^ |  |
|  | Icariin | *In vitro* | NB4  Cells | G1-phase ↑, S-phase ↓, Bcl-2 ↓, Bax ↑, Bax/Bcl-2 ↑ | Inhibiting proliferation, inducing apoptosis, and blocking cell cycle. | ^[140, 141]^ |  |
|  | Icariin | *In vitro* | HL-60, NB4 cells | Cleaved caspase-3 ↓, cleaved PARP ↓, MMP ↓, SOD ↓, GSH↓, ROS ↓ | Reducing cell viability, inducing apotosis, and increasing ATO-induced oxidative stress. | ^[142]^ |  |
|  | Icariin | *In vitro* | K562 cells | p-p85 ↑, p-Akt ↑, cleavage-caspse-3↑ | Inhibiting proliferation and inducing apoptosis. | ^[144]^ |  |
|  | Icariin | *In vivo* | DBA rats | Bcl-2 ↓, p-Akt ↓, Akt mRNA ↓, PTEN mRNA ↑ | Inhibiting proliferation. | ^[143]^ |  |
|  | Icariin | *In vivo* | MDS rats | Morbid hematopoiesis of bone marrow ↑, bone marrow cells ↓ | Inducing apotosis. | ^[145]^ |  |
|  | Icariin | *In vitro* | Raji, P3HR-1 cells | cleaved caspase-8 ↑, cleaved caspase-9 ↑, cleaved PARP ↑, Bcl-2 ↓, c-Myc ↓ | Inhibiting proliferation, inducing apoptosis, and blocking cell cycle. | ^[146]^ |  |
|  | Icariin | *In vitro* | K562, U937, P3H1 and Raji cells |  | Failing to inhibit Raji cell, and inhibiting K562, U937 and P3H1 cells growth. (IC_50_>100 ʯg/mL) | ^[147]^ |  |
|  | Icariin | *In vitro* | U266 cells | STAT3↓, p-JAK1↓, p-JAK2↓, and p-Src↓, Bcl- 2↓, Bcl-xl↓, Survivin↓, IAP-1↓, IAP-2↓, COX-2↓, VEGF↓, MMP-9↓ | Inhibiting cell proliferation and inducing apoptosis. | ^[151]^ |  |
|  | Icariin | *In vitro* | U266 and primary MM cells | Cyclin A↓, Cyclin B↓, CDK2↓, cyclin E↑, Bak↑, Bax↑, Bcl-xL↓, p-STAT3↓, p-JAK2↓, caspase 3↑, caspase 9↑, IL-6/ JAK2/STAT3 pathway↓ | Inhibiting cell proliferation and inducing apoptosis. | ^[150]^ |  |
|  | Icariin | *In vivo* | MM xenograft mouse models | IL-6↓, IgE↓, IL-6/ JAK2/STAT3 pathway↓ | Suppressing tumor growth. | ^[150]^ |  |
|  | Icariin | *In vitro* | MM CD138+, MM BMMNC, U266 and KM3 cells | Cleaved PARP(89KD)↑, Erk↑, JNK↑, c-jun↑ | Inhibiting cell proliferation and inducing apoptosis. | ^[152]^ |  |

**REFERENCE**

[1] SUN S, LIU L, TIAN X, et al. Icariin Attenuates High Glucose-Induced Apoptosis, Oxidative Stress, and Inflammation in Human Umbilical Venous Endothelial Cells [J]. Planta Med, 2019, 85(6): 473-82.

[2] HE C, WANG Z, SHI J. Pharmacological effects of icariin [J]. Adv Pharmacol, 2020, 87(179-203.

[3] ANGELONI C, BARBALACE M C, HRELIA S. Icariin and Its Metabolites as Potential Protective Phytochemicals Against Alzheimer's Disease [J]. Front Pharmacol, 2019, 10(271.

[4] SZABó R, RáCZ C P, DULF F V. Bioavailability Improvement Strategies for Icariin and Its Derivates: A Review [J]. Int J Mol Sci, 2022, 23(14):

[5] SONG L, CHEN X, MI L, et al. Icariin-induced inhibition of SIRT6/NF-κB triggers redox mediated apoptosis and enhances anti-tumor immunity in triple-negative breast cancer [J]. Cancer Sci, 2020, 111(11): 4242-56.

[6] WU Z M, XIANG Y R, ZHU X B, et al. Icariin represses the inflammatory responses and survival of rheumatoid arthritis fibroblast-like synoviocytes by regulating the TRIB1/TLR2/NF-kB pathway [J]. Int Immunopharmacol, 2022, 110(108991.

[7] ZENG Y, XIONG Y, YANG T, et al. Icariin and its metabolites as potential protective phytochemicals against cardiovascular disease: From effects to molecular mechanisms [J]. Biomed Pharmacother, 2022, 147(112642.

[8] WANG Z, WANG D, YANG D, et al. The effect of icariin on bone metabolism and its potential clinical application [J]. Osteoporos Int, 2018, 29(3): 535-44.

[9] SUNG H, FERLAY J, SIEGEL R L, et al. Global Cancer Statistics 2020: GLOBOCAN Estimates of Incidence and Mortality Worldwide for 36 Cancers in 185 Countries [J]. CA Cancer J Clin, 2021, 71(3): 209-49.

[10] NUSSINOV R, TSAI C J, JANG H. Anticancer drug resistance: An update and perspective [J]. Drug Resist Updat, 2021, 59(100796.

[11] NEOPTOLEMOS J P, KLEEFF J, MICHL P, et al. Therapeutic developments in pancreatic cancer: current and future perspectives [J]. Nat Rev Gastroenterol Hepatol, 2018, 15(6): 333-48.

[12] XU J Y, LIU F Y, LIU S X, et al. Plant-Derived Chinese Medicine Monomers on Ovarian Cancer via the Wnt/β-Catenin Signaling Pathway: Review of Mechanisms and Prospects [J]. J Oncol, 2021, 2021(6852867.

[13] WANG S, LONG S, DENG Z, et al. Positive Role of Chinese Herbal Medicine in Cancer Immune Regulation [J]. Am J Chin Med, 2020, 48(7): 1577-92.

[14] WANG Y, ZHANG Q, CHEN Y, et al. Antitumor effects of immunity-enhancing traditional Chinese medicine [J]. Biomed Pharmacother, 2020, 121(109570.

[15] ZHANG X, QIU H, LI C, et al. The positive role of traditional Chinese medicine as an adjunctive therapy for cancer [J]. Biosci Trends, 2021, 15(5): 283-98.

[16] XU W, LI B, XU M, et al. Traditional Chinese medicine for precancerous lesions of gastric cancer: A review [J]. Biomed Pharmacother, 2022, 146(112542.

[17] TAN H L, CHAN K G, PUSPARAJAH P, et al. Anti-Cancer Properties of the Naturally Occurring Aphrodisiacs: Icariin and Its Derivatives [J]. Front Pharmacol, 2016, 7(191.

[18] ZHANG C, SUI X, JIANG Y, et al. Antitumor effects of icaritin and the molecular mechanisms [J]. Discov Med, 2020, 29(156): 5-16.

[19] LIU Y, YANG H, XIONG J, et al. Icariin as an emerging candidate drug for anticancer treatment: Current status and perspective [J]. Biomed Pharmacother, 2023, 157(113991.

[20] ZHU F, REN Z. Icariin inhibits the malignant progression of lung cancer by affecting the PI3K/Akt pathway through the miR‑205‑5p/PTEN axis [J]. Oncol Rep, 2022, 47(6):

[21] RAWAL S, KHOT S, BORA V, et al. Surface-modified nanoparticles of docetaxel for chemotherapy of lung cancer: An intravenous to oral switch [J]. Int J Pharm, 2023, 636(122846.

[22] WEN Y M, LIANG Z A, XU Y B. Effects of icariin inhibiting PI3K/AKT pathway on the survival and metastasis of lung adenocarcinoma A549 cells. [J]. Chin J Immunol, 2020, 36(17): 2091-5.

[23] WU X, KONG W, QI X, et al. Icariin induces apoptosis of human lung adenocarcinoma cells by activating the mitochondrial apoptotic pathway [J]. Life Sci, 2019, 239(116879.

[24] RUILIAN Z, YING G, HONGMEI S, et al. Exploration of the Effect of Icariin on Nude Mice with Lung Cancer Bone Metastasis via the OPG/RANKL/RANK System [J]. Comput Math Methods Med, 2022, 2022(2011625.

[25] GONG W Y. Effect and mechanism of flavonoids from Scutellaria baicalensis Georgi on nicotine induced lung cancer progression. [D]; Fudan University, 2013.

[26] HAN L. Study on the mechanism of icariin enhancing miR-370 and down regulating PIM1 in inhibiting lung cancer. [D]; Jilin University, 2017.

[27] ZHU W S. Study on the mechanism of icariin inhibiting the proliferation of lung cancer A549 cells. [D]; Huazhong University of Science and Technology, 2013.

[28] WU J F, HE X D, XU W D, et al. Icariin reverses the metastatic phenotype of methotrexate resistant lung cancer A549 cells [J]. Tumour, 2009, 29(12): 1124-8.

[29] ZHOU X, ZHAO W H, ZHAO X. Evaluation of the efficacy and safety of Akoladine combined with conventional regimen in the treatment of advanced non-small cell lung cancer [J]. Journal of the People's Liberation Army Medical College, 2019, 40(03): 219-22.

[30] JI Y, ZHANG Z, HOU W, et al. Enhanced antitumor effect of icariin nanoparticles coated with iRGD functionalized erythrocyte membrane [J]. Eur J Pharmacol, 2022, 931(175225.

[31] SZYMONOWICZ K A, CHEN J. Biological and clinical aspects of HPV-related cancers [J]. Cancer Biol Med, 2020, 17(4): 864-78.

[32] JIANG G, WANG X, ZHOU Y, et al. TMTP1-Modified, Tumor Microenvironment Responsive Nanoparticles Co-Deliver Cisplatin and Paclitaxel Prodrugs for Effective Cervical Cancer Therapy [J]. Int J Nanomedicine, 2021, 16(4087-104.

[33] DU D B, LU W G, CAO C R, et al. Effect of icariin on apoptosis of cervical cancer TC-1 cells [J]. Progress in Modern Biomedicine, 2011, 11(04): 646-9.

[34] YU Z T, WANG M L, WANG J J. Study on the Effect of Paeonol Combined with Icariin on the Growth of Cervical Cancer Hela Cells by CXCR3-B and CXCL4. [J]. Guangdong Chemical Journal, 2021, 48(09): 123-4.

[35] HUANG S, XIE T, LIU W. Icariin inhibits the growth of human cervical cancer cells by inducing apoptosis and autophagy by targeting mTOR/PI3K/AKT signalling pathway [J]. J buon, 2019, 24(3): 990-6.

[36] LI C, YANG S, MA H, et al. Influence of icariin on inflammation, apoptosis, invasion, and tumor immunity in cervical cancer by reducing the TLR4/MyD88/NF-kappaB and Wnt/beta-catenin pathways [J]. Cancer Cell Int, 2021, 21(1): 206.

[37] SIEGEL R L, MILLER K D, FUCHS H E, et al. Cancer Statistics, 2021 [J]. CA Cancer J Clin, 2021, 71(1): 7-33.

[38] VISTAD I, BJøRGE L. Advanced endometrial cancer: New medical treatment options on the horizon [J]. Acta Obstet Gynecol Scand, 2023, 102(2): 128-9.

[39] ZHAO L M, PAN X M, SONG H Q, et al. Experimental study on icariin enhancing the killing sensitivity of CIK cells to B-MD-C1 (ADR + / +) [J]. Journal of Immunology, 2011, 27(02): 114-8.

[40] WANG G, YAN F, WANG Y, et al. Visual Sensing of β-Glucosidase From Intestinal Fungus in the Generation of Cytotoxic Icarisid II [J]. Front Chem, 2022, 10(919624.

[41] JAMMAL M P, LIMA C A, MURTA E F C, et al. Is Ovarian Cancer Prevention Currently Still a recommendation of Our Grandparents? [J]. Rev Bras Ginecol Obstet, 2017, 39(12): 676-85.

[42] ELYASHIV O, WONG Y N S, LEDERMANN J A. Frontline Maintenance Treatment for Ovarian Cancer [J]. Curr Oncol Rep, 2021, 23(8): 97.

[43] LI J W, WANG S Z, ZHAO F J. Icariin upregulates miR-519d expression and inhibits proliferation, migration and invasion of human SKOV3 ovarian cancer cells [J]. Advances in Anatomy, 2015, 21(05): 471-4+8.

[44] CHEN R, SU Y, LIU J. Icariin via Wnt/ β- Effects of catenin signaling pathway on the proliferation of ovarian cancer cell line CAOV3 [J]. Journal of Medical Research, 2019, 48(03): 44-9.

[45] GAO J, FU Y, SONG L, et al. Proapoptotic Effect of Icariin on Human Ovarian Cancer Cells via the NF-[Formula: see text]B/PI3K-AKT Signaling Pathway: A Network Pharmacology-Directed Experimental Investigation [J]. Am J Chin Med, 2022, 50(2): 589-619.

[46] HE Q. Effects of icariin on migration and invasion of human ovarian cancer SKOV3 cells [D]; Nanchang University, 2016.

[47] JIANG S Y, CHANG H, FAN D Y, et al. Inhibitory effect of icariin on malignant behavior of human ovarian cancer cell line [J]. Journal of Sichuan University (Medical Edition), 2018, 49(04): 530-4.

[48] WANG S, GAO J, LI Q, et al. Study on the regulatory mechanism and experimental verification of icariin for the treatment of ovarian cancer based on network pharmacology [J]. J Ethnopharmacol, 2020, 262(113189.

[49] LI J, JIANG K, ZHAO F. Icariin regulates the proliferation and apoptosis of human ovarian cancer cells through microRNA-21 by targeting PTEN, RECK and Bcl-2 [J]. Oncol Rep, 2015, 33(6): 2829-36.

[50] JIANG S, CHANG H, DENG S, et al. Icariin enhances the chemosensitivity of cisplatin‑resistant ovarian cancer cells by suppressing autophagy via activation of the AKT/mTOR/ATG5 pathway [J]. Int J Oncol, 2019, 54(6): 1933-42.

[51] ALHAKAMY N A, U A F, BADR-ELDIN S M, et al. Optimized Icariin Phytosomes Exhibit Enhanced Cytotoxicity and Apoptosis-Inducing Activities in Ovarian Cancer Cells [J]. Pharmaceutics, 2020, 12(4):

[52] WANG P, ZHANG J, XIONG X, et al. Icariin suppresses cell cycle transition and cell migration in ovarian cancer cells [J]. Oncol Rep, 2019, 41(4): 2321-8.

[53] FU Y, LIU H, LONG M, et al. Icariin attenuates the tumor growth by targeting miR-1-3p/TNKS2/Wnt/β-catenin signaling axis in ovarian cancer [J]. Front Oncol, 2022, 12(940926.

[54] BRAY F, FERLAY J, SOERJOMATARAM I, et al. Global cancer statistics 2018: GLOBOCAN estimates of incidence and mortality worldwide for 36 cancers in 185 countries [J]. CA Cancer J Clin, 2018, 68(6): 394-424.

[55] DESAI K, MCMANUS J M, SHARIFI N. Hormonal Therapy for Prostate Cancer [J]. Endocr Rev, 2021, 42(3): 354-73.

[56] LAFONTAINE M L, KOKOROVIC A. Cardiometabolic side effects of androgen deprivation therapy in prostate cancer [J]. Curr Opin Support Palliat Care, 2022, 16(4): 216-22.

[57] BUTTIGLIERO C, TUCCI M, VIGNANI F, et al. Chemotherapy-Induced Neutropenia and Outcome in Patients With Metastatic Castration-Resistant Prostate Cancer Treated With First-Line Docetaxel [J]. Clin Genitourin Cancer, 2018, 16(4): 318-24.

[58] ZHANG J. Effect of icariin on prostate cancer LNCaP cells and its AR related molecular mechanism [D]; Beijing University of Traditional Chinese Medicine, 2013.

[59] ZHANG W H, ZHANG W C, YU Y D. Effects of icariin on the activity, migration and invasion of prostate cancer cell lines [J]. Chinese Journal of Pathophysiology, 2017, 33(06): 1017-20.

[60] CHEN D S, HU X Y, CHEN S X, et al. Effect of icariin on androgen receptor signal pathway in orthotopic prostate cancer xenografts in SCID mice [J]. Chinese Journal of Comparative Medicine, 2018, 28(10): 49-54.

[61] CHEN S X, RAO H, CHEN D S. Effect of icariin on androgen receptor signal transduction pathway of androgen dependent prostate cancer in BALB/c-nu nude mice [J]. Journal of Changchun University of Traditional Chinese Medicine, 2018, 34(03): 436-8.

[62] HE H Q, RAO H, ZHENG J, et al. Effect of icariin on androgen receptor signal pathway in orthotopic prostate cancer transplantation model mice [J]. World Science and Technology - Modernization of Traditional Chinese Medicine, 2019, 21(04): 641-6.

[63] RAO H, WU F Y, HE H Q. Effects of icariin on fatty acid synthase and biological behavior of LNCaP cells in SCID mice with orthotopic prostate cancer [J]. Practical medicine and clinic, 2018, 21(05): 498-501.

[64] ZHANG Y, WEI Y, ZHU Z, et al. Icariin enhances radiosensitivity of colorectal cancer cells by suppressing NF-kappaB activity [J]. Cell Biochem Biophys, 2014, 69(2): 303-10.

[65] TIAN M, YANG S, YAN X. Icariin reduces human colon carcinoma cell growth and metastasis by enhancing p53 activities [J]. Braz J Med Biol Res, 2018, 51(10): e7151.

[66] ZHANG L, QIAO D W, DONG X Y, et al. Inhibitory effect and mechanism of icariin on proliferation and invasion of colon cancer [J]. Journal of Yangzhou University (Agriculture and Life Sciences Edition), 2019, 40(03): 81-5.

[67] KIM B, SEO J H, LEE K Y, et al. Icariin sensitizes human colon cancer cells to TRAIL‑induced apoptosis via ERK‑mediated upregulation of death receptors [J]. Int J Oncol, 2020, 56(3): 821-34.

[68] SHI D B, LI X X, ZHENG H T, et al. Icariin-mediated inhibition of NF-kappaB activity enhances the in vitro and in vivo antitumour effect of 5-fluorouracil in colorectal cancer [J]. Cell Biochem Biophys, 2014, 69(3): 523-30.

[69] HAO H, ZHANG Q, ZHU H, et al. Icaritin promotes tumor T-cell infiltration and induces antitumor immunity in mice [J]. Eur J Immunol, 2019, 49(12): 2235-44.

[70] SMYTH E C, NILSSON M, GRABSCH H I, et al. Gastric cancer [J]. Lancet, 2020, 396(10251): 635-48.

[71] ZHAO Q, CAO L, GUAN L, et al. Immunotherapy for gastric cancer: dilemmas and prospect [J]. Brief Funct Genomics, 2019, 18(2): 107-12.

[72] TIFFNER A, DERLER I. Molecular Choreography and Structure of Ca(2+) Release-Activated Ca(2+) (CRAC) and K(Ca2+) Channels and Their Relevance in Disease with Special Focus on Cancer [J]. Membranes (Basel), 2020, 10(12):

[73] LI S. Effects of Icariin on CaSR and expression of Survivin and RUNX3 genes in SGC-7901 cells with gastric cancer [D]; Gansu University of traditional Chinese Medicine, 2016.

[74] YIN Y, XU W, SONG Y, et al. Icariin Regulates the hsa_circ_0003159/eIF4A3/bcl-2 Axis to Promote Gastric Cancer Cell Apoptosis [J]. Evid Based Complement Alternat Med, 2022, 2022(1955101.

[75] ZHANG F, YIN Y, XU W, et al. Icariin inhibits gastric cancer cell growth by regulating the hsa_circ_0003159/miR-223-3p/NLRP3 signaling axis [J]. Hum Exp Toxicol, 2022, 41(9603271221097363.

[76] DUAN W F, SU J R, FU X M. Effect of Icariin on the proliferation of gastric cancer cell line SGC-7901 [J]. Chinese medicine guidelines, 2009, 7(24): 17-9.

[77] CHEN S H, MA T J, ZHANG Z H. Icariin inhibited proliferation, migration and invasion of MGC803 Cells by down regulating metastasis associated protein 2 [J]. Basic medicine and clinical, 2019, 39(11): 1587-91.

[78] WANG Y, DONG H, ZHU M, et al. Icariin exterts negative effects on human gastric cancer cell invasion and migration by vasodilator-stimulated phosphoprotein via Rac1 pathway [J]. Eur J Pharmacol, 2010, 635(1-3): 40-8.

[79] RIZVI S, WANG J, EL-KHOUEIRY A B. Liver Cancer Immunity [J]. Hepatology, 2021, 73 Suppl 1(Suppl 1): 86-103.

[80] MOKDAD A A, SINGAL A G, YOPP A C. JAMA PATIENT PAGE. Treatment of Liver Cancer [J]. Jama, 2016, 315(1): 100.

[81] LI J, XUAN S, DONG P, et al. Immunotherapy of hepatocellular carcinoma: recent progress and new strategy [J]. Front Immunol, 2023, 14(1192506.

[82] XU J W, YOU W W. Study on the effect and mechanism of icariin on human liver cancer HepG2 cells [J]. Journal of Integrated Traditional Chinese and Western Medicine in Hepatology, 2023, 33(01): 47-50.

[83] BI Y T, HUA J M, LIN J. Icariin passes through Akt/GSK3 β/ CDK pathway inhibits proliferation of CLC5 liver cancer cells [J]. Chinese Journal of Experimental Traditional Medical Formulae, 2022, 28(12): 96-102.

[84] ZHU Y H, HUANG L X, SHI C J. Effects of icariin on proliferation and apoptosis of liver cancer cell line SMMC-7721 [J]. Chinese Journal of General Surgery, 2012, 21(08): 968-72.

[85] LI W. The inhibitory effect of icariin combined with arsenic trioxide on human hepatocellular carcinoma [D]; Shandong University, 2014.

[86] WANG C Q, PENG X C. Study on the mechanism of icariin inhibiting the migration of HepG2 liver cancer cells [J]. Lishizhen Medicine and Materia Medica Research, 2011, 22(09): 2196-7.

[87] YANG J X, FICHTNER I, BECKER M, et al. Anti-proliferative efficacy of icariin on HepG2 hepatoma and its possible mechanism of action [J]. Am J Chin Med, 2009, 37(6): 1153-65.

[88] TANG J, ZHANG L, LI C L. Inhibition of icariin and baicalin combined with doxorubicin on the expression of APRIL in hepatocellular carcinoma cells and the growth of vascular endothelial cells [J]. Chinese Journal of Cancer Prevention and Treatment, 2009, 16(20): 1534-7.

[89] WANG Q, ZHANG L, MAO H T. Study on the inhibitory effect of traditional Chinese medicine icariin on the proliferation and immune escape of HepG2.2.15 liver cancer cells [J]. CHINESE JOURNAL OF IMMUNOLOGY, 2007, 10): 908-11.

[90] TANG J, LI C L, YANG S J. Icariin, baicalin and doxorubicin inhibit APRIL expression and reverse tumor immune escape in hepatocellular carcinoma cells [J]. Chinese Journal of Cancer Biotherapy, 2007, 06): 516-21.

[91] DING Y, YU B, ZHOU S, et al. Improvement of solubility and pharmacokinetic profile of hepatoprotector icariin through complexation with HP-γ-cyclodextrin [J]. Front Pharmacol, 2023, 14(1138686.

[92] LI J, SHEN S, LIU Z, et al. Synthesis and Structure-Activity Analysis of Icaritin Derivatives as Potential Tumor Growth Inhibitors of Hepatocellular Carcinoma Cells [J]. J Nat Prod, 2023, 86(2): 290-306.

[93] CAO Z, CHENG Y, WANG J, et al. HBP1-mediated transcriptional repression of AFP inhibits hepatoma progression [J]. J Exp Clin Cancer Res, 2021, 40(1): 118.

[94] LI H, LIU Y, JIANG W, et al. Icaritin promotes apoptosis and inhibits proliferation by down-regulating AFP gene expression in hepatocellular carcinoma [J]. BMC Cancer, 2021, 21(1): 318.

[95] XUE Z, ZHANG F, XU S, et al. Investigating the effect of Icaritin on hepatocellular carcinoma based on network pharmacology [J]. Frontiers in Pharmacology, 2023, 14(

[96] BAILLY C. Molecular and cellular basis of the anticancer activity of the prenylated flavonoid icaritin in hepatocellular carcinoma [J]. Chemico-Biological Interactions, 2020, 325(109124.

[97] FAN Y, LI S, DING X, et al. First-in-class immune-modulating small molecule Icaritin in advanced hepatocellular carcinoma: preliminary results of safety, durable survival and immune biomarkers [J]. BMC Cancer, 2019, 19(1): 279.

[98] SUN Y, LI Q, XU J-M, et al. A multicenter, single arm phase II trial of a small molecule immune-modulator icaritin: Safety, overall survival, immune dynamics, and PD-L1 expression in advanced hepatocellular carcinoma [J]. Journal of Clinical Oncology, 2018, 36(15_suppl): 4077-.

[99] BAIU I, VISSER B. Gallbladder Cancer [J]. Jama, 2018, 320(12): 1294.

[100] ROA J C, GARCíA P, KAPOOR V K, et al. Gallbladder cancer [J]. Nat Rev Dis Primers, 2022, 8(1): 69.

[101] ZHANG D C, LIU J L, DING Y B, et al. Icariin potentiates the antitumor activity of gemcitabine in gallbladder cancer by suppressing NF-κB [J]. Acta Pharmacol Sin, 2013, 34(2): 301-8.

[102] ZHANG H J, LI J X, ZHANG G B, et al. Effect of Epimedium on Migration and Adhesion of Human Gallbladder Cancer Cell Line GBC-SD [J]. Chinese and Foreign Health Abstracts, 2010, 7(14): 42-3.

[103] IKEDA G, YAMAMOTO S, KATO K. The safety of current treatment options for advanced esophageal cancer after first-line chemotherapy [J]. Expert Opin Drug Saf, 2022, 21(1): 55-65.

[104] JIN J, XU X, WANG F, et al. Second-line combination chemotherapy with docetaxel and nedaplatin for Cisplatin-pretreated refractory metastatic/recurrent esophageal squamous cell carcinoma [J]. J Thorac Oncol, 2009, 4(8): 1017-21.

[105] JI X, WANG C, LI J, et al. Icariin induces apoptosis of esophageal cancer cells in nude mice by enhancing Fas FasL expression activity [J]. Chongqing Medical, 2016, 45(12): 1608-11.

[106] GU Z F, ZHANG Z T, WANG J Y, et al. Icariin exerts inhibitory effects on the growth and metastasis of KYSE70 human esophageal carcinoma cells via PI3K/AKT and STAT3 pathways [J]. Environ Toxicol Pharmacol, 2017, 54(7-13.

[107] FAN C, YANG Y, LIU Y, et al. Icariin displays anticancer activity against human esophageal cancer cells via regulating endoplasmic reticulum stress-mediated apoptotic signaling [J]. Sci Rep, 2016, 6(21145.

[108] HAN S C. Effect and mechanism of Icariin on physiological activity of esophageal cancer stem cells [D]; Gansu University of traditional Chinese Medicine, 2019.

[109] MASTHAN K M, BABU N A, DASH K C, et al. Advanced diagnostic aids in oral cancer [J]. Asian Pac J Cancer Prev, 2012, 13(8): 3573-6.

[110] WANG F, GOUTTIA O G, WANG L, et al. PARP1 Upregulation in Recurrent Oral Cancer and Treatment Resistance

Radioiodine therapy in advanced differentiated thyroid cancer: Resistance and overcoming strategy [J]. Front Cell Dev Biol, 2021, 9(804962.

[111] SUN L, ZHANG J. Icariin inhibits oral squamous cell carcinoma cell proliferation and induces apoptosis via inhibiting the NF-κB and PI3K/AKT pathways [J]. Exp Ther Med, 2021, 22(3): 942.

[112] LEI K, MA B, SHI P, et al. Icariin Mitigates the Growth and Invasion Ability of Human Oral Squamous Cell Carcinoma via Inhibiting Toll-Like Receptor 4 and Phosphorylation of NF-κB P65 [J]. Onco Targets Ther, 2020, 13(299-307.

[113] LENIS A T, LEC P M, CHAMIE K, et al. Bladder Cancer: A Review [J]. Jama, 2020, 324(19): 1980-91.

[114] SEOK J, KWAK H J, KWAK Y, et al. Anti-oncogenic effects of dutasteride, a dual 5-alpha reductase inhibitor and a drug for benign prostate hyperplasia, in bladder cancer [J]. J Transl Med, 2023, 21(1): 129.

[115] BRAUSI M, ODDENS J, SYLVESTER R, et al. Side effects of Bacillus Calmette-Guérin (BCG) in the treatment of intermediate- and high-risk Ta, T1 papillary carcinoma of the bladder: results of the EORTC genito-urinary cancers group randomised phase 3 study comparing one-third dose with full dose and 1 year with 3 years of maintenance BCG [J]. Eur Urol, 2014, 65(1): 69-76.

[116] GAO M. Inhibitory effect of icariin on human bladder cancer T24 cells in vitro [D]; Hebei Medical University, 2010.

[117] KANG Z J, SUN J, ZHANG Y, et al. Effect of icariin on GRP78 gene expression in human bladder cancer BIU87 cells [J]. Journal of Traditional Chinese Medicine, 2013, 28(12): 1792-3.

[118] ROOF L, GEIGER J L. Clinical Utility of Cabozantinib in the Treatment of Locally Advanced or Metastatic Differentiated Thyroid Carcinoma: Patient Selection and Reported Outcomes [J]. Cancer Manag Res, 2023, 15(343-50.

[119] LIU Y, WANG J, HU X, et al. Radioiodine therapy in advanced differentiated thyroid cancer: Resistance and overcoming strategy [J]. Drug Resist Updat, 2023, 68(100939.

[120] ALSEN M, SINCLAIR C, COOKE P, et al. Endocrine Disrupting Chemicals and Thyroid Cancer: An Overview [J]. Toxics, 2021, 9(1):

[121] ZHENG C M, LIU X Z, LI Q L, et al. Study on icariin reversing the activity of bisphenol A in promoting thyroid cancer B-CPAP cells [J]. Chinese Journal of Otolaryngology Head and Neck Surgery, 2017, 52(06): 458-62.

[122] CHEN C, XIE L, REN T, et al. Immunotherapy for osteosarcoma: Fundamental mechanism, rationale, and recent breakthroughs [J]. Cancer Lett, 2021, 500(1-10.

[123] WANG Z, YANG L, XIA Y, et al. Icariin enhances cytotoxicity of doxorubicin in human multidrug-resistant osteosarcoma cells by inhibition of ABCB1 and down-regulation of the PI3K/Akt pathway [J]. Biol Pharm Bull, 2015, 38(2): 277-84.

[124] WANG Z D, WANG R Z, XIA Y Z, et al. Reversal of multidrug resistance by icaritin in doxorubicin-resistant human osteosarcoma cells [J]. Chin J Nat Med, 2018, 16(1): 20-8.

[125] LU P. Experimental study on the effect of icariin on human osteosarcoma cells [D]; Central South University, 2012.

[126] REN Y, ZHU F, LIU Z. Inhibitory effect of icariin on osteosarcoma cell proliferation via the Wnt/β-catenin signaling pathway [J]. Oncol Lett, 2018, 16(2): 1405-10.

[127] TAN L Q, MAI W X. Regulation of icariin on proliferation, apoptosis and migration of human osteosarcoma U2OS cells [J]. Journal of Medical Molecular Biology, 2019, 16(2): 132-6,42.

[128] RAUF A, IMRAN M, BUTT M S, et al. Resveratrol as an anti-cancer agent: A review [J]. Crit Rev Food Sci Nutr, 2018, 58(9): 1428-47.

[129] WU J, SONG T, LIU S, et al. Icariside II inhibits cell proliferation and induces cell cycle arrest through the ROS-p38-p53 signaling pathway in A375 human melanoma cells [J]. Mol Med Rep, 2015, 11(1): 410-6.

[130] WANG D, XU W, CHEN X, et al. Icariin induces cell differentiation and cell cycle arrest in mouse melanoma B16 cells via Erk1/2-p38-JNK-dependent pathway [J]. Oncotarget, 2017, 8(59): 99504-13.

[131] DONGYE Z, WU X, WEN Y, et al. Icaritin and intratumoral injection of CpG treatment synergistically promote T cell infiltration and antitumor immune response in mice [J]. Int Immunopharmacol, 2022, 111(109093.

[132] LI X, SUN J, HU S, et al. Icariin Induced B16 Melanoma Tumor Cells Apoptosis, Suppressed Tumor Growth and Metastasis [J]. Iran J Public Health, 2014, 43(6): 847-8.

[133] WANG M, GAO H, LI W, et al. Icariin and its metabolites regulate lipid metabolism: From effects to molecular mechanisms [J]. Biomed Pharmacother, 2020, 131(110675.

[134] ROGERS H W, WEINSTOCK M A, HARRIS A R, et al. Incidence estimate of nonmelanoma skin cancer in the United States, 2006 [J]. Arch Dermatol, 2010, 146(3): 283-7.

[135] AGGARWAL P, KNABEL P, FLEISCHER A B, JR. United States burden of melanoma and non-melanoma skin cancer from 1990 to 2019 [J]. J Am Acad Dermatol, 2021, 85(2): 388-95.

[136] WU J, ZUO F, DU J, et al. Icariside II induces apoptosis via inhibition of the EGFR pathways in A431 human epidermoid carcinoma cells [J]. Mol Med Rep, 2013, 8(2): 597-602.

[137] BARBUI T, THIELE J, GISSLINGER H, et al. The 2016 WHO classification and diagnostic criteria for myeloproliferative neoplasms: document summary and in-depth discussion [J]. Blood Cancer J, 2018, 8(2): 15.

[138] LI Y Y. Observation on the differentiation of K562 leukemia cells induced by icaritin hydrate and its mechanism [D]; Central South University, 2009.

[139] GE L F, DONG Z J, JIANG G S, et al. Effects of Icariin on acute promyelocytic leukemia cells in vitro and in vivo [J]. Journal of Cancer Prevention and Treatment, 2001, 06): 622-4.

[140] SONG F, GAO S J, ZHANG Y W, et al. Effect of icariin on proliferation and apoptosis of NB4 leukemia cell line and its mechanism [J]. Journal of Jilin University (Medical Edition), 2015, 41(06): 1181-5.

[141] SONG F. Inhibitory effect and mechanism of icariin on proliferation and apoptosis of NB4 leukemia cell line [D]; Jilin University, 2012.

[142] WANG Z, ZHANG H, DAI L, et al. Arsenic Trioxide and Icariin Show Synergistic Anti-leukemic Activity [J]. Cell Biochem Biophys, 2015, 73(1): 213-9.

[143] WANG L, KE H. Experimental Study on the Regulation of Icariin on PI3K/Akt Signaling Pathway in Acute Lymphocytic Leukemia [J]. Journal of Emergency in Traditional Chinese Medicine, 2018, 27(06): 1000-3+11.

[144] PAN L P, YUAN W. Study on icariin promoting apoptosis of leukemia K562 cells [J]. Leukemia · Lymphoma, 2016, 25(06): 340-3.

[145] FENG X Y, YAN L P, YANG Y. Effect of icariin on apoptosis of bone marrow cells in rats with myelodysplastic syndrome [J]. Shizhen Traditional Chinese Medicine Traditional Chinese Medicine, 2007, 18(12): 3070-2.

[146] LI Z J, YAO C, LIU S F, et al. Cytotoxic effect of icaritin and its mechanisms in inducing apoptosis in human burkitt lymphoma cell line [J]. Biomed Res Int, 2014, 2014(391512.

[147] LIN C C, NG L T, HSU F F, et al. Cytotoxic effects of Coptis chinensis and Epimedium sagittatum extracts and their major constituents (berberine, coptisine and icariin) on hepatoma and leukaemia cell growth [J]. Clin Exp Pharmacol Physiol, 2004, 31(1-2): 65-9.

[148] OWENS B. Outlook: Multiple myeloma [J]. Nature, 2020, 587(7835): S55.

[149] MOREAU P, SAN MIGUEL J, SONNEVELD P, et al. Multiple myeloma: ESMO Clinical Practice Guidelines for diagnosis, treatment and follow-up [J]. Ann Oncol, 2017, 28(suppl_4): iv52-iv61.

[150] ZHU S, WANG Z, LI Z, et al. Icaritin suppresses multiple myeloma, by inhibiting IL-6/JAK2/STAT3 [J]. Oncotarget, 2015, 6(12): 10460-72.

[151] JUNG Y Y, LEE J H, NAM D, et al. Anti-myeloma Effects of Icariin Are Mediated Through the Attenuation of JAK/STAT3-Dependent Signaling Cascade [J]. Front Pharmacol, 2018, 9(531.

[152] LI Z J. Study on the anti multiple myeloma effect of icariin and its molecular mechanism [D]; Central South University, 2011.

[153] NUSSBAUMER S, BONNABRY P, VEUTHEY J L, et al. Analysis of anticancer drugs: a review [J]. Talanta, 2011, 85(5): 2265-89.

[154] GUICHARD N, GUILLARME D, BONNABRY P, et al. Antineoplastic drugs and their analysis: a state of the art review [J]. Analyst, 2017, 142(13): 2273-321.

[155] BUKOWSKI K, KCIUK M, KONTEK R. Mechanisms of Multidrug Resistance in Cancer Chemotherapy [J]. Int J Mol Sci, 2020, 21(9):

[156] KNEZEVIC C E, CLARKE W. Cancer Chemotherapy: The Case for Therapeutic Drug Monitoring [J]. Ther Drug Monit, 2020, 42(1): 6-19.

[157] GHOSH S. Cisplatin: The first metal based anticancer drug [J]. Bioorg Chem, 2019, 88(102925.

[158] DEVITA V T, JR., CHU E. A history of cancer chemotherapy [J]. Cancer Res, 2008, 68(21): 8643-53.

[159] ZHAI K, MAZURAKOVA A, KOKLESOVA L, et al. Flavonoids Synergistically Enhance the Anti-Glioblastoma Effects of Chemotherapeutic Drugs [J]. Biomolecules, 2021, 11(12):

[160] YUAN J Y, TONG Z Y, DONG Y C, et al. Research progress on icariin, a traditional Chinese medicine extract, in the treatment of asthma [J]. Allergol Immunopathol (Madr), 2022, 50(1): 9-16.

[161] TEO Y L, CHEONG W F, CAZENAVE-GASSIOT A, et al. Pharmacokinetics of Prenylflavonoids following Oral Ingestion of Standardized Epimedium Extract in Humans [J]. Planta Med, 2019, 85(4): 347-55.

[162] ZHANG C, XU C, GAO X, et al. Platinum-based drugs for cancer therapy and anti-tumor strategies [J]. Theranostics, 2022, 12(5): 2115-32.

[163] PFISTER D G. The just price of cancer drugs and the growing cost of cancer care: oncologists need to be part of the solution [J]. J Clin Oncol, 2013, 31(28): 3487-9.

[164] YANG Q Z, ZHANG Y, WANG Y J, et al. Icariin activates CaMK Ⅱ JNK pathway to inhibit the survival and metastasis of human non-small cell lung cancer A549 cells [J]. Public Health in China, 2020, 36(07): 1014-9.

[165] DI S, FAN C, YANG Y, et al. Activation of endoplasmic reticulum stress is involved in the activity of icariin against human lung adenocarcinoma cells [J]. Apoptosis, 2015, 20(9): 1229-41.

[166] GU C M, ZHAN Y R, CHEN Y F. Analysis of Icariin in Herba Epimedii Drug containing Serum and Study on Its Antitumor Effect in Vitro [J]. Journal of Guangdong Pharmaceutical University, 2020, 36(04): 499-503.
